# Orientational Jahn–Teller Isomerism in the Dark‐Stable State of Nature's Water Oxidase

**DOI:** 10.1002/anie.202103425

**Published:** 2021-05-06

**Authors:** Maria Drosou, Georgia Zahariou, Dimitrios A. Pantazis

**Affiliations:** ^1^ Inorganic Chemistry Laboratory National and Kapodistrian University of Athens, Panepistimiopolis Zografou 15771 Greece; ^2^ Institute of Nanoscience & Nanotechnology, NCSR “Demokritos” Athens 15310 Greece; ^3^ Max-Planck-Institut für Kohlenforschung Kaiser-Wilhelm-Platz 1 45470 Mülheim an der Ruhr Germany

**Keywords:** bioinorganic chemistry, computational chemistry, electronic structure, EPR spectroscopy, photosynthesis

## Abstract

The tetramanganese–calcium cluster of the oxygen‐evolving complex of photosystem II adopts electronically and magnetically distinct but interconvertible valence isomeric forms in its first light‐driven oxidized catalytic state, S_2_. This bistability is implicated in gating the final catalytic states preceding O−O bond formation, but it is unknown how the biological system enables its emergence and controls its effect. Here we show that the Mn_4_CaO_5_ cluster in the resting (dark‐stable) S_1_ state adopts orientational Jahn–Teller isomeric forms arising from a directional change in electronic configuration of the “dangler” Mn^III^ ion. The isomers are consistent with available structural data and explain previously unresolved electron paramagnetic resonance spectroscopic observations on the S_1_ state. This unique isomerism in the resting state is shown to be the electronic origin of valence isomerism in the S_2_ state, establishing a functional role of orientational Jahn–Teller isomerism unprecedented in biological or artificial catalysis.

## Graphical abstract

The dark‐stable resting state of the biological oxygen‐evolving complex is shown to accommodate a rare type of functionally important Mn^III^ orientational Jahn–Teller isomerism that is identified as the electronic origin of subsequent valence isomerism in the catalytic cycle of water oxidation.

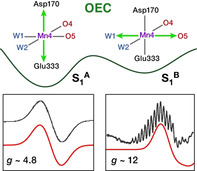

## Introduction

**Figure 1:**
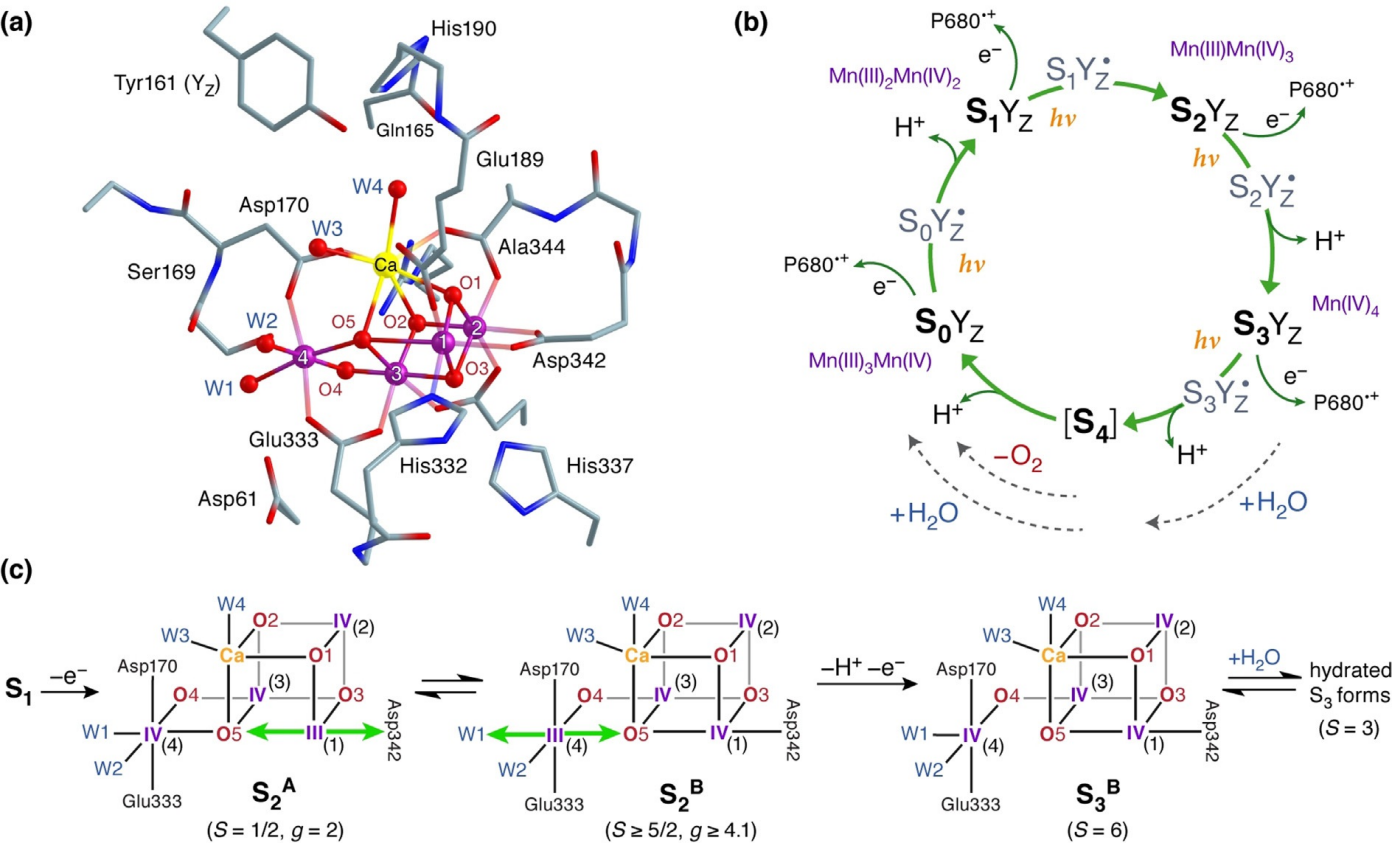
a) Structure of the oxygen‐evolving complex suggested by crystallography (PDB ID: 5B66).^[13]^ b) The S‐state cycle of the OEC, illustrating the light‐induced one‐electron oxidation steps coupled to proton release, the uptake of substrate water molecules, and O_2_ evolution. Electron transfer from the OEC to the P680 radical cation of the PSII reaction center is mediated by a redox‐active tyrosine (Tyr161 or Y_Z_) giving rise to metalloradical S_*i*_Y_Z_^.^ intermediates. c) Spectroscopically consistent S_2_ state valence isomers **S_2_** ^**A**^ and **S_2_** ^**B**^, which gate progression to a high‐spin water‐unbound form of the S_3_ state.^[27]^ The orientation of Jahn–Teller elongation for the Mn^III^ ion in each S_2_ isomer is indicated with green arrows.

The oxygen‐evolving complex (OEC) is a unique system common to all oxygenic photosynthetic organisms that catalyzes the four‐electron oxidation of water, powered by light‐driven charge separation at the reaction center of photosystem II (PSII). The Mn_4_CaO_5_ cluster at the heart of the OEC serves both as the charge accumulator that sequentially stores the four required oxidizing equivalents through a succession of states S_*i*_ (*i*=0–4), and as the catalyst that activates substrate water molecules enabling O−O bond formation in the transient S_4_ state (Figure 1). The essential structural details of the OEC have been painstakingly refined over the years.[^1^, ^2^, ^3^, ^4^, ^5^] Protein crystallography has focused primarily on the dark‐stable (resting) S_1_ state of the enzyme[^6^, ^7^, ^8^, ^9^] yielding structural models of increasing reliability, although uncertainty persists about important details.[^5^, ^10^, ^11^, ^12^, ^13^, ^14^, ^15^] In the parallel efforts of spectroscopic and quantum chemical studies to provide experimentally consistent atomistic models for other catalytic states and intermediates, a most intriguing outcome has been the role of polymorphism in the OEC. Specifically, the S_2_ state of the Mn_4_CaO_5_ cluster with oxidation states Mn^III^Mn^IV^_3_ is known to exhibit two types of EPR signal,[^16^, ^17^, ^18^, ^19^] at *g*=2 (multiline signal, spin *S*=1/2) and *g*≥4.1 (*S*≥5/2). Several explanations for this phenomenon have been considered (see Supporting Information), but the most well‐supported scenario suggests that the signals arise from two valence isomeric forms (referred to as **S_2_** ^**A**^ and **S_2_** ^**B**^) that differ in the position of the unique Mn^III^ ion, Mn1 in **S_2_** ^**A**^ and Mn4 in **S_2_** ^**B**^, respectively (Figure 1).[^20^, ^21^, ^22^] The two valence isomers, which are close in energy and interconvertible,[^20^, ^22^, ^23^] have distinct spin states and spectroscopic properties that correspond to the two known types of EPR signal[^16^, ^17^, ^18^, ^19^] for the S_2_ state: the **S_2_** ^**A**^ with spin *S=*1/2 and *g*=2, and the **S_2_** ^**B**^ with *S*≥5/2 and *g*≥4.1. Orientation dependence of the *g*=4.1 signal confirms the location of the Mn^III^ ion at the outer Mn4 ion and the orientation of the zero‐field splitting (ZFS) *z*‐axis along the Mn4‐W1 vector.^[24]^

The emergence of valence isomers in the S_2_ state may be crucial for controlling access to the last experimentally observable S_3_ state.[^25^, ^26^, ^27^] The valence isomers are part of a gating mechanism in which the majority form (**S_2_** ^**A**^) is more easily deprotonated yet the minority form (**S_2_** ^**B**^) can be more easily oxidized to an all‐Mn^IV^ species before binding an additional water molecule.^[26]^ This water‐unbound form of the S_3_ state (Figure 1) was recently identified by EPR spectroscopy on spinach PSII as a high‐spin (*S*=6) species with high effective anisotropy,^[27]^ in contrast to water‐bound S_3_ intermediates that have intermediate‐spin *S*=3 ground states.[^27^, ^28^, ^29^, ^30^, ^31^, ^32^] The details of the S_2_‐S_3_ transition and the precise composition of the heterogeneous S_3_ state are subjects of intense current research efforts because of their direct implications for the mechanism of the final unresolved steps of O_2_ formation and evolution. On the other hand, despite the functional relevance of the S_2_ state valence isomerism for catalytic progression, the fundamental question of how the enzyme enables and controls its emergence remains entirely open. Although structural heterogeneity in the resting S_1_ state of the OEC has been previously considered,[^12^, ^13^, ^33^] it remains unclear which intrinsic feature enables the cluster to generate distinct valence isomers upon oxidation. The present work answers this question with the aid of quantum chemical and electron paramagnetic resonance (EPR) analysis of the S_1_ state, by establishing the existence and demonstrating the functional role of orientational Jahn–Teller isomerism in the OEC.

## Results and Discussion

### Orientational Jahn–Teller Isomers in the Dark‐Stable State of the OEC

**Figure 2:**
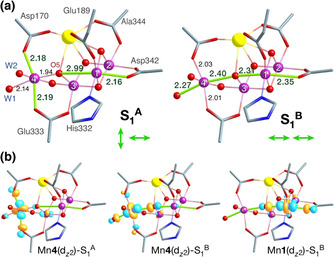
a) Core geometries of S_1_ models **S_1_** ^**A**^ and **S_1_** ^**B**^, with selected optimized bond lengths shown for comparison and with indication of the Jahn–Teller axis orientations (in green). b) Canonical molecular orbitals with Mn^III^ $d_{z^{2}}$ character, showing the distinct orientation of the Mn4 $d_{z^{2}}$ orbital in each isomer.

Based on insights from previous modeling of the S_2_ state,[^34^, ^35^, ^36^] large quantum mechanical cluster models were created starting from the coordinates of the 5B66 (monomer A) crystal structure^[13]^ for exploring possible forms of the S_1_ state. Geometry optimizations were carried out with density functional theory for various spin configurations, with the Mn4 ligand W2 in both the hydroxo and aquo forms, and the O5 bridge in both the oxo and hydroxo forms (Table S1). Two different motifs of the inorganic core within the same coordination environment and valence distribution III‐IV‐IV‐III for the Mn1‐Mn2‐Mn3‐Mn4 cluster were identified (Figure 2). The structures of the distinct S_1_ forms indicate that they both contain a Mn1(III) ion with its (pseudo‐)Jahn–Teller axis of tetragonal elongation oriented along Asp342‐O5, but differ in the Jahn–Teller distortion of the Mn4(III) ion. In the form labeled **S_1_** ^**A**^ the Jahn–Teller elongation axis of Mn4 lies along the Asp170‐Glu333 vector, perpendicular to the Jahn–Teller axis of Mn1, whereas in the **S_1_** ^**B**^ isomer the Jahn–Teller elongation axis of Mn4 is collinear to that of Mn1 and oriented along the W1‐O5 vector. The axial Mn‐O elongations are so prominent that the orientations of Jahn–Teller axes can be read directly from the geometric parameters, but the electronic structure also perfectly mirrors the above description. The Kohn–Sham molecular orbitals (Figure 2 b) show that both terminal Mn^III^ ions of **S_1_** ^**A**^ and **S_1_** ^**B**^ have occupied $d_{z^{2}}$ orbitals that are σ‐antibonding with respect to the ligands aligned with the structurally identified Jahn–Teller axes.

**Figure 3:**
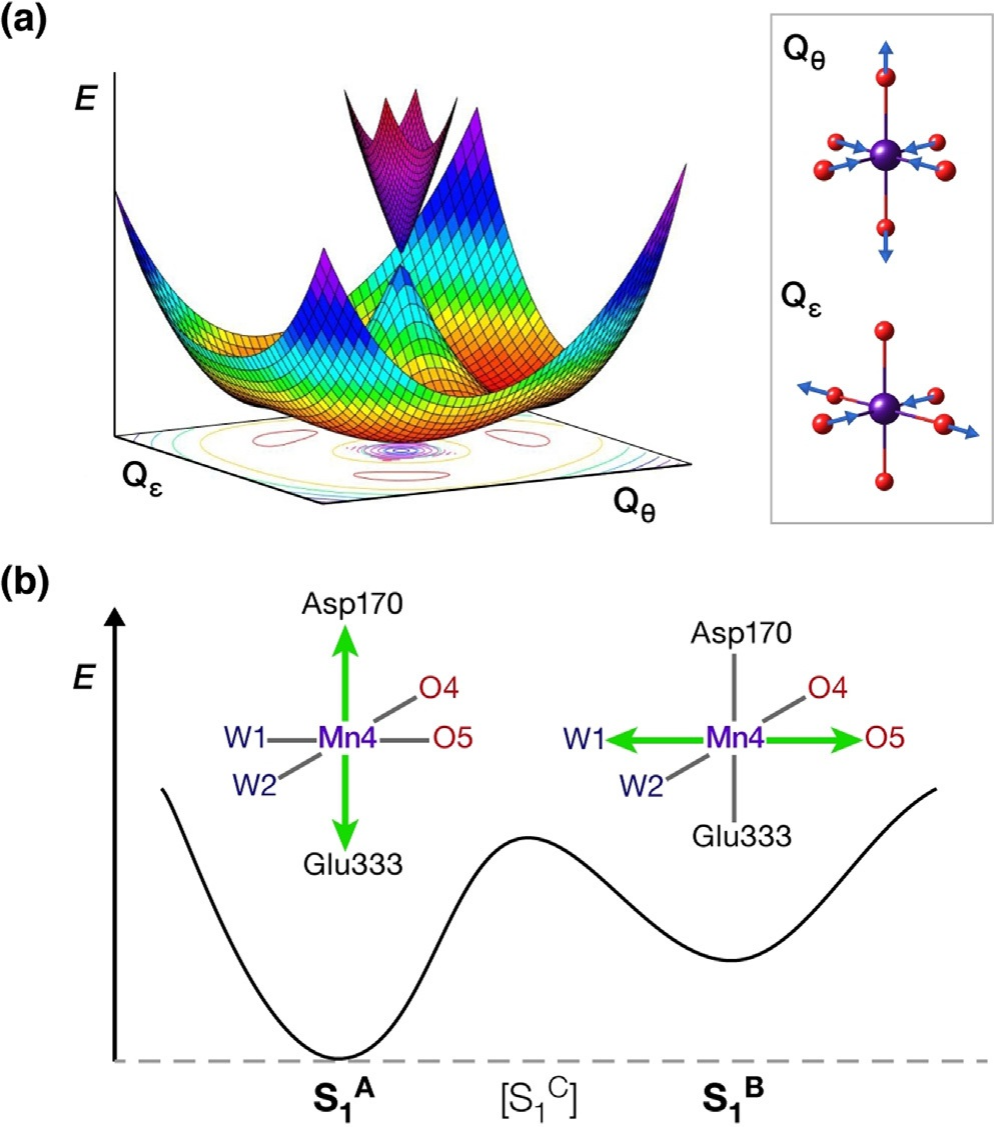
a) Potential energy surface resulting from the quadratic *E*⊗*e* coupling for an idealized octahedral Mn^III^ ion with illustration of the two types of distortion (see also Figure S1). b) Schematic depiction of the partial potential energy trough cross‐section suggested to survive for the orientational Jahn–Teller S_1_ isomers identified here with respect to configurations of the terminal Mn4(III) ion. Jahn–Teller elongation axes depicted with green arrows.

The adiabatic potential energy surface for an octahedral homoleptic Mn^III^ high‐spin d^4^ system (*E*⊗*e* coupling problem) in the space defined by the tetragonal and orthorhombic distortion coordinates Q_θ_ and Q_*ϵ*_ has three minima at the level of quadratic interaction corresponding to the three directions of tetragonal distortions (Figure S1 and Figure 3 a). In heteroleptic, asymmetrically coordinated Mn^III^ complexes, environment anisotropy lifts the degeneracy of the three minima and typically results in localization and alignment of the (pseudo) Jahn–Teller axis along a single “privileged” direction. In the present case the **S_1_** ^**A**^ and **S_1_** ^**B**^ are assigned as local Jahn–Teller minima with respect to the Mn4 ion, whereas a third isomer, **S_1_** ^**C**^, was also located and assigned as the transition state that connects them (Figure S2). In **S_1_** ^**C**^ the Mn4 ion has a singly occupied $d_{x^{2}-y^{2}}$ orbital and a Jahn–Teller compression axis along the Mn4‐W2 bond, similar to a model previously discussed by Paul et al.^[37]^

The coordination sphere of the Mn4 ion of the OEC is extremely unusual in enabling, despite the asymmetric ligation, the presence of two distinct minima with the Mn4 elongation axis either along Glu333‐Asp170 (**S_1_** ^**A**^) or W1‐O5 (**S_1_** ^**B**^). A factor enabling this type of isomerism may be the unusual ligation mode of D1‐Asp170, a residue that bridges Mn4 and Ca but interacts with the latter in a lopsided fashion that allows bifurcated H‐bonding interaction with CP43‐Arg357 (Figure 1). The third possible orientation for Mn4 and alternative Jahn–Teller orientations for Mn1 could not be located even when starting the self‐consistent Kohn–Sham procedure with appropriately manipulated orbital guesses and geometries.

In terms of energetics, isomer **S_1_** ^**A**^ is computed to be 3.2 kcal mol^−1^ lower than **S_1_** ^**B**^. Conversion of **S_1_** ^**A**^ to **S_1_** ^**B**^ occurs via **S_1_** ^**C**^, which is 5.3 kcal mol^−1^ higher than **S_1_** ^**A**^ and where the octahedron becomes compressed along the W2‐Mn4‐O4 axis. In view of the nature of the problem and the limitations of DFT it is inadvisable to treat these energy values as quantitative predictions. The present description is not an exact theoretical treatment of the vibronic coupling problem and only highlights obvious analogies between the formal problem and the actual situation in the OEC as approximated by DFT. Nevertheless, the computed energies suggest that interchange of the different minima can be inhibited at cryogenic temperatures used for EPR spectroscopic studies of catalytic intermediates, which is important for our structural interpretation of the corpus of S_1_ state EPR spectroscopy developed in the following.

The importance of the Jahn–Teller effect for shaping the geometric structure of Mn^III^‐containing clusters is recognized,[^34^, ^38^, ^39^, ^40^, ^41^, ^42^, ^43^] but orientational Jahn–Teller isomerism is unexpected for fully heteroleptic Mn^III^ centers. Contrary to spin‐state interconversions and redox isomerism, Jahn–Teller isomerism is rare in metalloenzymatic catalysis,^[44]^ while specifically the orientation switching described here is without precedence in biology. A possibly unique analogue in synthetic inorganic chemistry is the report by Christou and co‐workers of two Jahn–Teller axis orientations for the same Mn^III^ ion in a Mn12 single molecule magnet, with each orientational Jahn–Teller isomer displaying distinct relaxation behavior.[^45^, ^46^]

### Correlation with Experimental Structural Information

All S_1_ models discussed above are consistent with available crystallographic data on the OEC, giving root mean square deviation (RMSD) of less than 0.17 Å with respect to the highest‐resolution crystallographic model of Photosystem II (Table S1). **S_1_** ^**A**^ Mn‐Mn distances are closer to the corresponding crystallographic and X‐ray absorption spectroscopy (EXAFS) derived values than **S_1_** ^**B**^, which is attributed to the elongation of Mn3‐Mn4 distance and decrease of the Mn1‐Mn3 distance that take place as the Mn4‐O5 distance increases (Figure S3). The isomers reported here resemble forms of the cluster reported by Narzi et al.^[12]^ and we similarly find that a *combination* of the **S_1_** ^**A**^ and **S_1_** ^**B**^ isomeric forms provides the best agreement with EXAFS and X‐ray crystallography structural data (Table S1 and Figure S3). Given that **S_1_** ^**C**^ has intermediate values between **S_1_** ^**A**^ and **S_1_** ^**B**^ it would also appear in better agreement with structural data than each of the minima individually, but the energetics and nature of the stationary points support **S_1_** ^**A**^/**S_1_** ^**B**^ heterogeneity rather than predominance of the **S_1_** ^**C**^ form. S_1_ state heterogeneity combined with a percentage of the S_0_ state,^[12]^ in which O5 is protonated and Mn3 is reduced to Mn^III^ compared to the S_1_ state,[^15^, ^34^, ^47^, ^48^, ^49^] best rationalize the X‐ray free electron laser crystallographic model of PSII and the unusual apparent position of the O5 bridge.^[9]^ However, the heterogeneity described here is unlikely to be discernible by any experimental structural method at presently achievable levels of resolution.

The existence of the orientational Jahn–Teller isomers **S_1_** ^**A**^ and **S_1_** ^**B**^ in the S_1_ state provides a unique yet straightforward interpretation of Fourier transform infrared (FTIR) spectroscopic studies that documented spontaneous changes in carboxylate vibrational modes during dark adaptation of the S_1_ state of the OEC.^[50]^ Possible causes were suggested to include changes in carboxylate ligation to Mn, changes in secondary structure, and/or changes in polarity attributed to an unspecified isomer of the S_1_ state. The isomerism proposed here accounts for the FTIR observations because rotation of the Jahn–Teller axis of Mn4 directly affects the carboxylate Asp170 and Glu333 ligands by toggling the antibonding character of the respective Mn4−O bonds.

### Magnetism and Electron Paramagnetic Resonance Spectroscopy

Pairwise exchange coupling constants for structures **S_1_** ^**A**^ and **S_1_** ^**B**^ (Figure S4) were computed with broken‐symmetry density functional theory using TPSSh, an established functional for magnetic properties of manganese systems.[^51^, ^52^] In both structures Mn1 is strongly antiferromagnetically coupled with Mn2 and weakly ferromagnetically coupled with Mn3. Mn2 and Mn3 interact ferromagnetically and Mn1 shows a weak antiferromagnetic interaction with Mn4. **S_1_** ^**A**^ is diamagnetic with a low lying triplet first excited state, whereas **S_1_** ^**B**^ has an *S*=3 ground state with a quartet first excited state. Differences between the S_1_ models arise from *J*_34_, which is strongly antiferromagnetic when the Mn4‐O5 distance is short, but weakens rapidly as the Mn4‐O5 distance increases (Figure S4b).

Most compelling is the connection of the present models with EPR spectroscopy, which provides rich and selective information on the electronic structure of the OEC. The S_1_ state is typically discussed as a single entity in structural studies, but observations from EPR spectroscopy have always pointed toward heterogeneity. The first reported EPR signal of the S_1_ state was a featureless signal around *g*=4.8,[^53^, ^54^] observed in spinach. A different signal at *g*≈12 with resolved Mn hyperfine interactions was later reported for cyanobacterial OEC.^[55]^ This signal could also be induced in spinach by removal of two extrinsic PSII proteins,^[56]^ showing that the spectroscopic response of the OEC in the S_1_ state is sensitive to small structural perturbations. Dexheimer and Klein suggested that the state giving rise to the S_1_ *g*=4.8 signal converts to the S_2_ *g*=2 multiline state but does not correlate with the S_2_ *g*=4.1 signal,^[53]^ while Gregor and Britt showed that the S_2_ *g*=4.1 signal preferentially decays to the S_1_ multiline centered at *g*≈12.^[57]^ Sioros et al. supported that the S_1_Y_Z_^.^ component with the characteristic EPR signal at *g*=2.035 advances to the S_2_ multiline, while a 26 G wide signal advances to the *g*=4.1 conformation.^[58]^ EPR investigations of the *g*=2.035 component of the S_1_Y_Z_^.^ at X‐band and W‐band frequencies revealed that the major features of the spectra are due to the interaction of the *S*=1 state of the Mn_4_ cluster with the *S*=1/2 spin of Y_Z_^.^.^[59]^ These observations are consistent with the idea that the S_1_ state consists of two isomers that are the predecessors of the *g*=2 or *g*=4.1 forms of the S_2_ state. The two EPR signals described above remain without explicit atomistic explanation and even their common origin has been questioned.^[55]^ Here we show that they are both attributable to the S_1_ state and have a one‐to‐one correspondence with the Jahn–Teller isomers described above.

**Figure 4:**
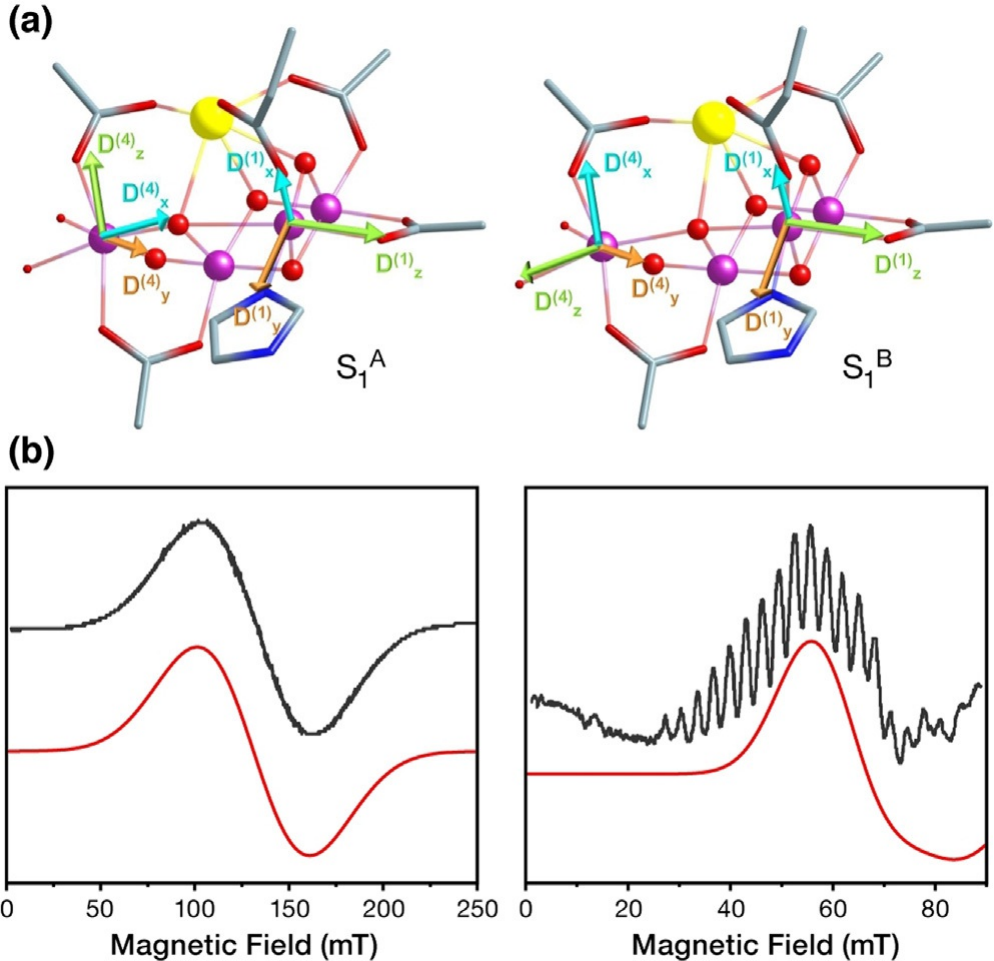
a) Orientation of principal axes of *D*_SOC_ for Mn^III^ ions in the **S_1_** ^**A**^ and **S_1_** ^**B**^ Jahn–Teller isomers, computed by multireference L‐CASCI calculations. b) Simulations of the two types of S_1_ EPR signal. Left: Simulation of EPR signal at *g*_eff_≈4.8 using *S*=1; acceptable combinations of *D* and *E*/*D* parameters are shown in Figure S5. Right: Simulation of the S_1_ EPR signal centered at *g*_eff_≈12, using *S*=3, *D*=6.67 cm^−1^, *E*/*D*=0.048.

To examine possible connections between the **S_1_** ^**A**^/**S_1_** ^**B**^ Jahn–Teller isomerism and available EPR data, we calculated local zero field splitting tensors (ZFS) of Mn1(III) and Mn4(III) ions using the multireference local complete active space configuration interaction (L‐CASCI) approach.^[60]^ The asymmetry of valence electronic configuration of Mn^III^ is connected to a pronounced Jahn–Teller distortion that produces sizable site anisotropy, contrary to the small site anisotropy (on the order of 0.3 cm^−1^) of Mn^IV^ ions. Consequently, Mn1(III) and Mn4(III) largely determine the total anisotropy of the complex. Calculated on‐site axial and rhombic anisotropy parameters are presented in Table S2 and orientations of the principal axes of the onsite *D*_SOC_ for each Mn^III^ ion are depicted in Figure 4 a. The |*D*| values of all Mn^III^ ions are ca. 3 cm^−1^, which indicates that perturbations on octahedral symmetry have limited impact on the axial part of the local anisotropy. Differences between the coordination environment of Mn1 and Mn4 are reflected in the rhombic ZFS values (*E*/*D*), as Mn1 has lower rhombicity than Mn4 due to higher asymmetry of the Mn4 coordination sphere. Additionally, the $D_{z}^{\left(i\right)}$ component of each Mn^III^ ion is collinear with its Jahn–Teller axis.

According to the computed ladder of spin eigenstates, **S_1_** ^**A**^ is diamagnetic and the observed signal derives from a low‐lying magnetically excited state with *S*=1, while **S_1_** ^**B**^ has an *S*=3 ground state and the first excited state is likely not populated at low temperatures. Based on the computed local ZFS tensors and the ground state spin configurations predicted by the pairwise exchange coupling constants, we calculated total ZFS tensors for structures **S_1_** ^**A**^ and **S_1_** ^**B**^ (see Supporting Information) and further examined whether the aforementioned spin configurations of the S_1_ isomeric forms and their ZFS parameters reproduce the experimental X‐band EPR spectra in parallel mode. The S_1_ EPR spectrum at *g*_eff_≈4.8 is indeed reproduced for *S*=1 (Figure 4 b, left) with a range of *D* and *E*/*D* parameters shown in Figure S5 that agree with the computed ones. It is noted that this signal cannot be described with *E*/*D*<0.17. This is consistent with the rhombic symmetry expected for **S_1_** ^**A**^ owing to the presence of the Jahn–Teller axis of each Mn^III^ at the *xy*‐plane of the other. In addition, a *S*=3 configuration with *E*/*D*=0.048 and *D* very close to the calculated value of 6.7 cm^−1^ reproduces the experimental derivative around *g*_eff_≈12 (Figure 4 b, right). The hyperfine structure of the multiline signal can additionally be reproduced using an effective hyperfine coupling constant for all Mn ions (Figure S6).

The present results establish that the Jahn–Teller isomers provide a simple and direct interpretation of both observed EPR signals and hence form a basis for revisiting and reinterpreting a large body of experimental spectroscopic work on a new atomistic basis.

### Mechanistic Implications for Catalytic Progression

The most important implication of Jahn–Teller isomerism in the S_1_ state is the functional role in the emergence of valence isomerism in the S_2_ state. The S_1_ isomers differ in a crucial electronic structure feature that is relevant for catalytic progression to the next state. The Mn1 $d_{z^{2}}$ orbital is lower in energy in **S_1_** ^**A**^ because the Mn1‐O5 antibonding interaction is minimized by movement of O5 away from Mn1 to form the Mn4‐O5 bond. As the Mn1‐O5 distance decreases, the Mn1 $d_{z^{2}}$ orbital is destabilized and the Mn4‐centered $d_{z^{2}}$ orbital is similarly stabilized in **S_1_** ^**B**^. The relative stabilities between the Mn1 and Mn4 valence orbitals suggest that oxidation of **S_1_** ^**A**^ involves oxidation of Mn4, leading to the oxidation state distribution III‐IV‐IV‐IV in the S_2_ state (i.e. the **S_2_** ^**A**^ model), whereas oxidation of **S_1_** ^**B**^ favors oxidation of Mn1, leading to the **S_2_** ^**B**^ model with a pendant Mn4(III) ion that retains its Jahn–Teller axis oriented towards O5.

**Figure 5:**
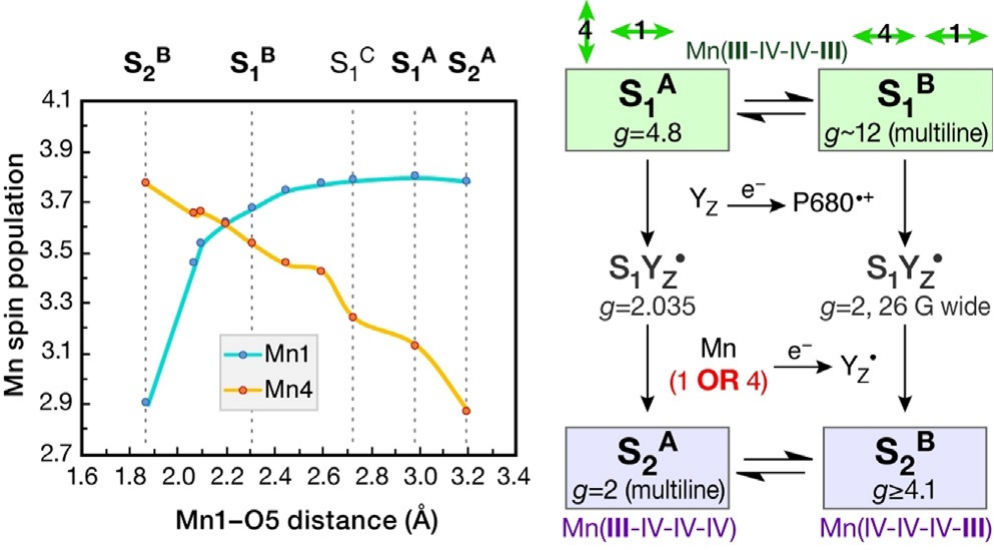
Left: Correlation of Mn1 (blue line) and Mn4 (orange line) Mulliken spin populations with the Mn1‐O5 distance for one‐electron oxidized S_1_ models derived from constrained geometry optimizations. Right: Correspondence between S_1_ and S_2_ isomer structures, showing how orientational Jahn–Teller isomerism in the dark‐stable S_1_ state of the OEC leads upon oxidation to distinct oxidation state distributions in the S_2_ state. The scheme indicates known EPR signals of the two states and metalloradical S_1_Y_Z_^.^ intermediates,^[58]^ showing how the isomeric forms accommodate spectroscopic observations into one coherent structural interpretation.

Figure 5 describes the changes in the Mn1 and Mn4 spin populations of the oxidized forms of S_1_ as a function of Mn1‐O5 bond length. The Mn4 spin population (orange line) reflects the aforementioned Mn4 $d_{z^{2}}$ stabilization as O5 moves toward Mn1, while the Mn1 line (blue) shows the concurrent Mn1 $d_{z^{2}}$ destabilization. According to the calculated spin populations, removal of an electron from **S_1_** ^**A**^ indeed takes place from Mn4, directly connecting the oxidized forms of **S_1_** ^**A**^ with **S_2_** ^**A**^. One‐electron oxidized **S_1_** ^**B**^ has a spin population of 3.67 on Mn1 and of 3.53 on Mn4. As shown in Figure 5, a Mn1/Mn4 spin population inversion in the oxidized form of S_1_ occurs at a Mn1‐O5 distance of 2.2 Å, which thus connects the oxidized form of **S_1_** ^**B**^ with **S_2_** ^**B**^. In conclusion, the emergence of valence isomers in the S_2_ state is a direct consequence of orientational Jahn–Teller isomerism in the dark‐stable S_1_ state of the enzyme. The diagram in Figure 5 summarizes our suggestion for the correspondence of EPR observations with Jahn–Teller or valence isomeric forms in the S_1_ and S_2_ states of the OEC.

In addition to structural and spectroscopic implications, orientational Jahn–Teller isomerism in the S_1_ state offers a new perspective for the analysis of substrate exchange kinetics.^[61]^ The position of O5 is most affected by the Jahn–Teller reorientation at Mn4, being at bonding distance to Mn4 in **S_1_** ^**A**^ but at non‐bonding distance to either of the terminal Mn ions in **S_1_** ^**Β**^. If O5 is a substrate,[^62^, ^63^] then it can be surmised that it would exchange faster in the **S_1_** ^**Β**^ model, positioned along the Jahn–Teller axes of both Mn1 and Mn4, as opposed to the **S_1_** ^**A**^ model, where it is more strongly bound to Mn4.

## Conclusion

Despite the asymmetric coordination sphere of the pendant manganese ion in the dark‐stable S_1_ state, the OEC of PSII accommodates stabilization of two distinct minima that correspond to different orientations of the Mn4(III) Jahn–Teller axis of tetragonal elongation. The two isomers provide a structure‐based interpretation of spectroscopic observations relating to the known EPR heterogeneity of the S_1_ state and of the S_1_‐S_2_ intermediates, because they fully rationalize the combined effect of local anisotropies in producing distinct EPR signals. Crucially, the locus of oxidation is different for each Jahn–Teller minimum and hence oxidation of each S_1_ isomer leads to a distinct oxidation state distribution in the S_2_ state. This explains how valence isomerism arises in the S_2_ state, a phenomenon implicated in control of substrate access in the later stages of the catalytic cycle. The connection of orientational Jahn–Teller isomerism with redox isomerism suggests that the natural system may have leveraged a unique electronic feature of the Mn^III^ ion to evolve an unprecedented mechanism for enabling and utilizing electronic structure fluxionality as an element of catalytic control.

## Conflict of interest

The authors declare no conflict of interest.

## Supporting information

As a service to our authors and readers, this journal provides supporting information supplied by the authors. Such materials are peer reviewed and may be re‐organized for online delivery, but are not copy‐edited or typeset. Technical support issues arising from supporting information (other than missing files) should be addressed to the authors.

Supplementary
