# Supplementary material for: Orientational Jahn–Teller Isomerism in the Dark‐Stable State of Nature's Water Oxidase
Source: Angew Chem Int Ed Engl. 2021 May 6;60(24):13493–9. doi: 10.1002/anie.202103425 (PMC8252073; doi:10.1002/anie.202103425)
Supplement: Supplementary file 1 — Supplementary [file ANIE-60-13493-s001.pdf]

Angewandte Chemie
Eine Zeitschrift der Gesellschaft Deutscher Chemiker

Supporting Information

Orientational Jahn–Teller Isomerism in the Dark-Stable State of Nature's Water Oxidase

*Maria Drosou, Georgia Zahariou, and Dimitrios A. Pantazis**

anie_202103425_sm_miscellaneous_information.pdf

Supporting Information

Contents

1. Methodology and computational details 2
2. Jahn-Teller isomerism 3
3. S_1-state optimized structures 3
4. Structural analysis 5
5. Pairwise exchange coupling constants 6
6. Available EPR data 6
7. Local ZFS parameters 7
8. Implications for the nature of the S_2 state 11
9. References 13
10. Cartesian coordinates of the presented models 15

1. Methodology and computational details

Construction and optimization of the S_1-state models. All calculations reported in this work were carried out with the ORCA program package.[1] OEC models are based on the XRD model of PSII at 1.9 Å resolution[2] and contain all first-sphere residues of the Mn_4CaO_5 core (water derived ligands W1–W4 and amino acids His332, Glu189, Asp342, Ala344, CP43-Glu354, Asp170, and Glu333), second-sphere residues that hydrogen-bond to the inorganic core or its first-sphere residues, several vicinal water molecules and the D1-Tyr161–His190 pair, along with complete backbone loops where necessary. Backbone constraints on peripheral peptide bonds and methyl groups were applied in order to preserve the effect of the protein matrix on the complex. Geometry optimizations were performed with the BP86 functional.[3-4] The ZORA-SVP basis sets were used for hydrogen and carbon atoms while the ZORA-TZVP basis sets were used for all other atoms.[5-7] The RI approximation was used with the fully decontracted Coulomb fitting auxiliary SARC/J basis sets. Increased integration grids (Grid4 in ORCA convention) and tight SCF convergence criteria were used throughout. The electrostatic influence of the environment was simulated using the conductor-like polarizable continuum model (CPCM) assuming a dielectric constant of ε=6.[8]

Calculation of pairwise exchange coupling constants. The magnetic coupling in the S_1-state models was investigated with the broken-symmetry density functional theory (DFT) methodology (BS-DFT), using the hybrid meta-GGA TPSSh functional[9] for single-point calculations of different spin configurations, with the chain-of-spheres (RIJCOSX) approximation[10] to exact exchange. Increased integration grids (Grid5 and GridX7 in ORCA convention) and tight SCF convergence criteria were used. The resulting overdetermined system of equations is solved by singular value decomposition to yield pairwise exchange coupling constants, J_{ij}. The complete energy ladder of spin eigenstates was calculated through diagonalization of the Heisenberg Hamiltonian:

$$\hat{H} = -2 \sum_{i<j} J_{ij} \hat{\boldsymbol{S}}_i \hat{\boldsymbol{S}}_j \qquad \text{eq. 1}$$

where S_i and S_j are the local spins of magnetic centers i and j, and J_{ij} the isotropic exchange coupling constant between centers i and j.

Calculation of local ZFS parameters. For the calculation of the on-site zero-field splitting (ZFS) parameters molecular orbitals were obtained from a spin-averaged restricted open-shell Hartree−Fock (SAHF) calculation,[11] following localization as proposed by Foster and Boys,[12] in order to assign each orbital to an individual magnetic center. The orbitals were subsequently recanonicalized and used to calculate the spin orbit coupling (SOC) contribution to the local ZFS of each Mn(III) center by performing a local configuration interaction calculation (L-CASCI)[13] in the space defined by the fourteen electrons and twenty d orbitals of the four Mn ions of the OEC. The excited states with local Mn(III) spin states S=2 and S=1 were considered when calculating the ZFS values, corresponding to total (cluster) spin multiplicities of 15 and 13, respectively.

EPR simulations. The EPR spectra were simulated with the Easyspin program[14] using the following Spin Hamiltonian:

$$\hat{H}_0 = g\beta\vec{B}\cdot\vec{S} + D\left[\vec{S}_z^2 - \frac{1}{3}S(S+1)\right] + E\left(\vec{S}_x^2 - \vec{S}_y^2\right) \qquad \text{eq. 2}$$

where the first term is the Zeeman interaction and the second and third terms represent the zero field interaction, where D and E are the axial and rhombic zero field splitting parameters, respectively.

2. Jahn-Teller isomerism

For an idealized octahedral Mn(III) high-spin d^4 system the doubly degenerate *E* electronic state couples with the doubly degenerate *e* normal mode that is defined by the two distortion coordinates that are conventionally assigned as Q_θ and Q_ε and define a tetragonal (elongation or compression) and an orthorhombic distortion of the molecular frame. Consideration of the linear terms in the $E\otimes e$ coupling introduces *radial* dependence resulting in an adiabatic potential energy surface characterized by a continuous minimum-energy circular trough, the well-known "Mexican hat" potential. This is modified by introduction of *angular* dependence via inclusion of quadratic terms: three distinct minima appear along the trough at $\varphi = 0$ and $\pm 2\pi/3$, an effect described as "warping of the hat".[15] The minima represent tetragonal distortions (typically elongations in the case of high-spin Mn(III) ions, i.e. $Q_\theta > 0$) along the three cubic axes of an octahedron (Figure S1). The height of the barriers determines the phenomenological characterization of a given system as statically or dynamically distorted.

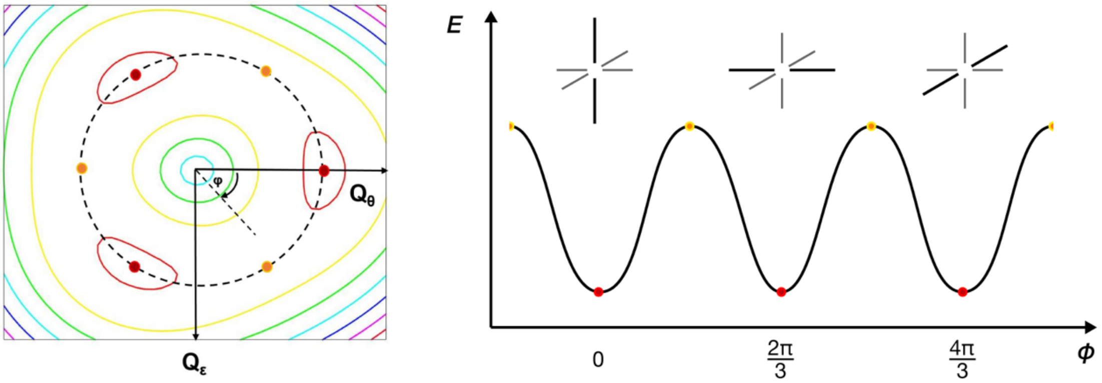

Figure S1. Potential energy plane diagram (left) and section across the circular "trough" (right) along the angle φ, showing the emergence of three minima (red circles) and maxima (orange circles) arising from non-zero quadratic terms in the Ee problem. The minima here correspond to differently oriented tetragonal elongations.

For octahedral high-spin Mn(III) complexes, with very rare exceptions (for example, refs [16-17]) the Jahn–Teller effect is associated with pronounced axial elongation, which can be so strong that one of the ligands dissociates. According to our calculations, the Mn1 Jahn-Teller axis in the dark-stable S_1 state of the OEC is always oriented along O5-Mn1-Asp342, thus the main text discussion is limited to a description in terms of a mononuclear Mn4-centered subsystem, for which a part of the above described surface is still identifiable.

3. S_1-state optimized structures

Different initial oxidation state distributions and electron configurations were set up and subsequently relaxed variationally towards electronically and geometrically optimized minima. All optimizations eventually led to only one low-lying oxidation state distribution with the two high-spin (d^4 electronic configuration) Mn(III) ions at the terminal positions of the cluster, Mn1 and Mn4, as clearly indicated by Mulliken spin populations of 3.8−4.0 for Mn(III) ions and 2.8−3.0 for Mn(IV) (high-spin d^3) ions. This is in agreement with previous assignments.[18-22] Different spin configurations during geometry optimization end up in the different Jahn-Teller isomers. In the main text the structures with the W2 in its hydroxo form are presented, but the Jahn-Teller isomerism is also observed when W2 is fully protonated to H_2O (all models presented in Table S1). When O5 is protonated every spin configuration gives a structure with parallel Mn1 and Mn4 Jahn-Teller axes. In structures $\mathbf{S_1^E}$ and $\mathbf{S_1^F}$ W2 is in the aquo form and $\mathbf{S_1^E}$ has

perpendicular Jahn-Teller Mn1 and Mn4 axes, while **S_1^F** has collinear Jahn-Teller Mn1 and Mn4 axes, similar to **S_1^A** and **S_1^B** forms.

Table S1. S_1-state optimized structures key structural parameters, spin configuration imposed during optimization and calculated relative energies. The RMSD values with respect to the 5B66-A crystallographic structure are presented, $RMSD^{22atoms}$ for a 22-atom fragment including the Mn_4CaO_5 core and first coordination sphere atoms and the $RMSD^{Mn}$ for the four Mn atoms only.

	O5, W2	Mn1-Mn2	Mn1-Mn3	Mn1-Mn4	Mn2-Mn3	Mn3-Mn4	Mn1-Ca	Mn2-Ca	Mn3-Ca	Mn4-Ca	Mn1-O5	Mn1-Asp342
S_1^A	O5=O, W2=OH	2.76	3.34	4.91	2.80	2.75	3.60	3.43	3.50	3.75	2.99	2.16
S_1^B	O5=O, W2=OH	2.76	3.07	4.70	2.78	2.87	3.41	3.43	3.46	3.83	2.31	2.35
S_1^C	O5=O, W2=OH	2.78	3.25	4.82	2.79	2.79	3.52	3.44	3.47	3.75	2.73	2.22
S_1^D	O5=OH, W2=OH	2.78	3.25	5.25	2.79	3.03	3.58	3.47	3.55	4.21	2.46	2.19
S_1^E	O5=O, W2=H_2O	2.76	3.38	4.99	2.80	2.72	3.63	3.42	3.53	3.81	3.13	2.13
S_1^F	O5=O, W2=H_2O	2.76	3.05	4.81	2.77	2.90	3.43	3.42	3.45	3.92	2.32	2.32

	Mn1-O1	Mn1-His332	Mn1-O3	Mn1-Glu189	Mn4-O5	Mn4-W1	Mn4-Asp170	Mn4-Glu333	Mn4-O4	Mn4-W2	RMSD 22 atoms	RMSD Mn atoms	Spin conf	ΔE[a], kcal mol^{-1}
S_1^A	1.85	2.06	1.92	1.93	1.94	2.14	2.18	2.19	1.91	1.88	0.17	0.055	abab	0.0
S_1^B	1.87	2.06	1.92	1.94	2.40	2.27	2.03	2.01	1.89	1.87	0.16	0.102	baaa	3.2
S_1^C	1.86	2.05	1.93	1.93	2.11	2.23	2.11	2.10	1.91	1.87	0.15	0.053	aabb	5.3
S_1^D	1.87	2.05	1.96	1.92	2.83	2.15	1.95	1.97	1.90	1.89	0.22	0.137	aaaa	16.0
S_1^E	1.85	2.05	1.92	1.92	1.88	2.07	2.14	2.17	1.85	2.07	0.17	0.075	abab	0.0
S_1^F	1.87	2.07	1.92	1.92	2.51	2.23	1.97	1.96	1.82	2.03	0.16	0.008	baaa	4.9

[a] Structures {S_1^A, S_1^B, S_1^C} and {S_1^D, S_1^E, S_1^F} have the same number of protons and electrons.

As presented in Table S1, using the BP86 optimized geometries in the respective spin configurations, the most stable of the three isomers discussed in the present work is **S_1^A**, followed by **S_1^B** (3.2 kcal mol^{-1}) and **S_1^C** (5.3 kcal mol^{-1}). Similar energy differences are obtained using different functionals with the same BP86 geometries. It is noted that with some functionals the relative stability of **S_1^A** and **S_1^B** may be reversed, but **S_1^C** remains higher than both, in accordance with the description of the Jahn–Teller potential energy surface discussed in this work. For example, using the popular B3LYP* functional (15% exact exchange) **S_1^A** is 1.7 kcal mol^{-1} and **S_1^C** is 3.1 kcal mol^{-1} higher than **S_1^B**. Using a larger, more polarized basis set (the def2 versions of the ZORA all-electron basis sets) the differences are negligible (with B3LYP*, **S_1^A** is 2.0 kcal mol^{-1} and **S_1^C** is 3.4 kcal mol^{-1} higher than **S_1^B**). Such small energy differences are well within the intrinsic uncertainty of the method and the uncertainty related to the structural definition of the models. They can be viewed, at most, as confirming the energetic proximity of the isomeric forms, which is consistent with the fact that minor perturbations in the protein environment result in preference of one EPR signal over the other.

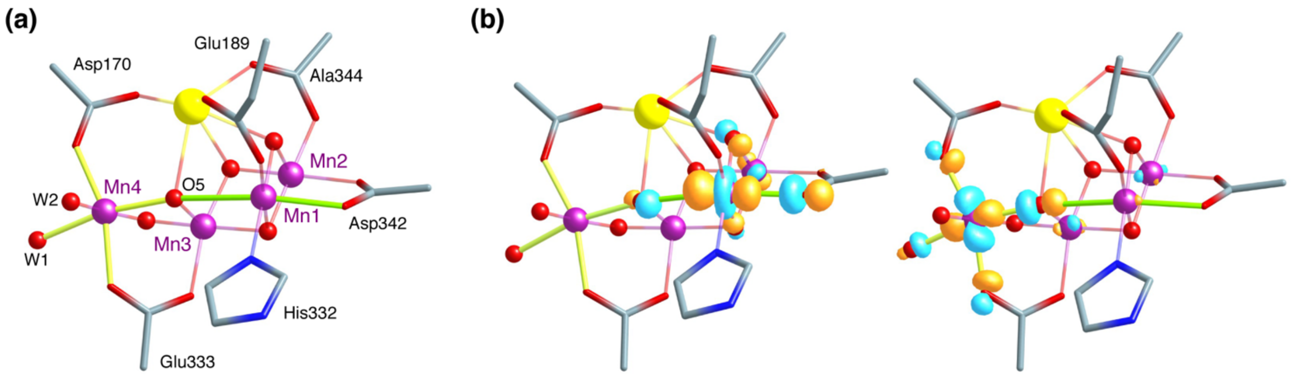

Figure S2. a) Schematic depiction of core geometry of optimized $\mathbf{S_1^C}$ model with the Jahn-Teller elongation axis of Mn1(III) in green and the Jahn-Teller elongated Mn4-O equatorial bonds (perpendicular to the Jahn-Teller compression axis along W2-Mn4-O4) in lime green. **b)** Canonical molecular orbitals corresponding to the singly occupied Mn1(III) d_{z^2} orbital (left) and Mn4(III) $\mathrm{d}_{x^2-y^2}$ orbital (right).

4. Structural analysis

The geometric differences of the isomeric forms described above are localized, therefore the Jahn–Teller isomers have high mutual structural similarity. 22-atom cores of models $\mathbf{S_1^A}$, $\mathbf{S_1^B}$ and $\mathbf{S_1^C}$ have root mean square deviation (RMSD) of less than 0.17 Å with respect to the 5B66 crystallographic values. EXAFS studies showed that there are two Mn−Mn distances at ~2.76 Å, one Mn−Mn distance at ~2.77 Å, and one Mn−Mn distance at ~3.28 Å in the S_1 state.[23-26] Therefore, $\mathbf{S_1^A}$ and $\mathbf{S_1^C}$ forms are in equally good agreement with EXAFS-derived Mn–Mn distances. Their RMSD for the Mn positions is only 0.055 Å and 0.053 Å, respectively, when compared to the X-ray values. Given the limited information content of EXAFS in the range of Mn–O distances,[27] the proposed isomers are in principle indistinguishable by EXAFS, particularly at high temperatures.[23] As the Mn4-O5 distance increases, the calculated Mn-Mn distances begin to deviate from experimental values, because the Mn3–Mn4 distance increases with concurrent reduction of the Mn1–Mn3 distance. EXAFS also indicated three Mn–Ca distances between 3.4 Å and 3.5 Å and one around 3.9 Å,[28] which is consistent with the longer Mn4–Ca distance of the $\mathbf{S_1^B}$ structure. Notably, the Mn4–Mn3 distance of each structure correlates with the Mn4–O5 distance, according to the equation R (Mn3–Mn4) = 0.25 * R (Mn4-O5) + 2.27 (Figure S3), which has also been observed by Yamaguchi and coworkers, on structures of the S_1 state derived from high-spin B3LYP QM/MM optimizations.[29-30]

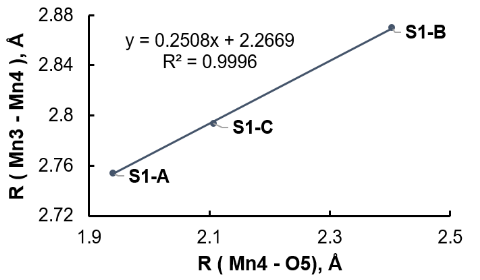

Figure S3. Correlation of the Mn4-O5 distance with the Mn3-Mn4 distance.

In the S_2 state, the O5 is found at non-bonding distance from the Mn(III) ion of each structure in the direction of its Jahn–Teller elongation axis, bound to Mn4(IV) in the $\mathbf{S_2^A}$ form in which Mn1 is Mn(III), and to Mn1(IV) in the $\mathbf{S_2^B}$ form where Mn(III) shifts to Mn4. In the $\mathbf{S_1^B}$ model that most resembles the $\mathbf{S_2^B}$ state

the Jahn–Teller axes of the terminal Mn(III) ions are aligned in the same way as each individual Mn(III) ion in either of the S_2 states, i.e. collinear and pointing towards O5. The analogy with the S_2 state is limited by the fact that the S_1 Jahn–Teller isomers presented here have the same Mn oxidation state distribution, and hence the electronic origin of heterogeneity in the S_1 state is fundamentally distinct from the valence isomerism of the S_2 state.

5. Pairwise exchange coupling constants

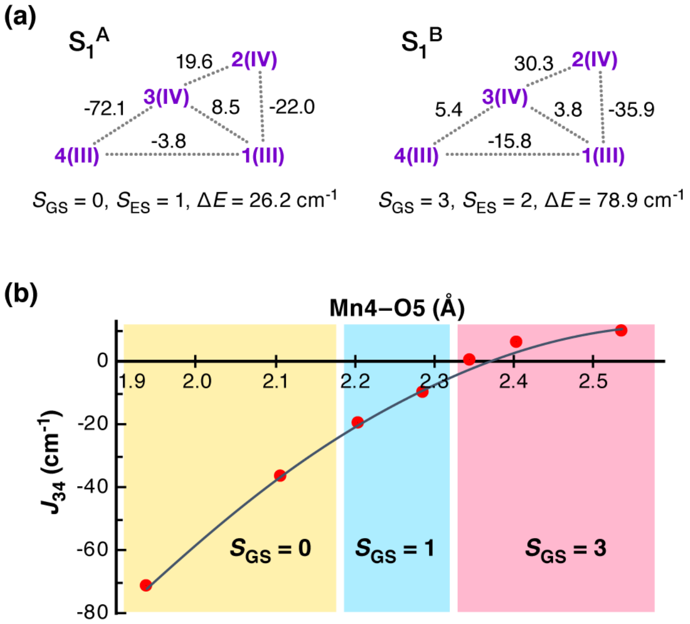

Figure S4. a) Mn oxidation states, nearest-neighbor pairwise exchange coupling constants (cm^{-1}) and relative energies of the ground (S_{GS}) and first excited (S_{ES}) spin states for the two S_1 isomers. Complete exchange coupling data for all reported models are provided in Table S2. **b)** Magnetostructural correlation of the Mn3-Mn4 exchange coupling J_{34} with the Mn4-O5 distance. The correlation fits well (R2 = 0.997) with the quadratic relationship $y = -182.64x^2 + 956.52x - 1240.7$. Shaded areas correspond to the different values adopted by the total ground state spin.

Table S2. Calculated exchange coupling constants, spin of the ground (S_{GS}) and first excited (S_{ES}) states, and their energy separation (in cm^{-1}) for the three Jahn-Teller isomers

	Pairwise exchange coupling constants, J (cm^{-1})						Magnetic states		
	J_{12}	J_{13}	J_{14}	J_{23}	J_{24}	J_{34}	S_{GS}	S_{EC}	ΔE
**S_1^A**	-21.98	8.47	-3.83	19.56	0.25	-72.10	0	1	26.2
**S_1^B**	-35.89	3.85	-15.81	30.23	-0.52	5.38	3	2	78.9
**S_1^C**	-21.16	9.92	-9.12	23.06	-0.37	-36.90	0	1	15.7

6. Available EPR data

Studies of metalloradical states created upon oxidation of the redox-active tyrosine residue (Y_Z) that mediates electron transfer between the OEC and the charge separation site of photosystem II ("split" $S_1Y_Z^{\bullet}$ signals) showed that spectroscopic heterogeneity develops during the S_1–S_2 transition, leaving open the possibility of unspecified heterogeneity in the S_1 state itself.[31-36] Ideas on the nature of this

hypothetical heterogeneity included the presence of different spin states or resting versus active forms,[37-38] of isomers with different valence and/or protonation states,[35, 39] and forms with different proximal hydrogen bonding networks.[40] However, none of these ideas has found support from independent experimental evidence or from theoretical modeling based on recent crystallographic models. The Jahn–Teller isomerism described here suggests that both signals may be attributable to the manganese cluster in the S_1 state.

The S_1-state g = 4.8 signal intensity increases with temperature and the temperature dependence shows that the signal can be assigned to an S = 1 excited state, lying 2.5 K above an S = 0 ground state.[41] By contrast, the S_1-state multiline g~12 signal amplitude increases with decreased temperature in a Curie law ($1/T$) behavior, which demonstrates that the signal arises from an integer spin ground state or a very low-lying excited state.[42-43] The two observed EPR signals (g = 4.8 and g = 12) are consistent with the idea of two stable forms of the S_1 state whose relative stability can be affected by second-sphere perturbations,[44] and which are associated with different EPR signals that are observable under different conditions. In attempts to correlate structure with spectroscopy, synthetic Mn4 complexes with $Mn(III)_2Mn(IV)_2$ oxidation states and g values that reproduce either of the S_1 state signals have been reported.[45-46] Of direct relevance is a recent analysis by Paul et al.[47] on the spectroscopic features of a structural model of the OEC reported by Zhang et al.[46] In that case EPR simulations of the synthetic OEC model g = 12 signal and the g = 4.8 OEC signal showed that they could be modeled as arising from S = 1 spin systems with effective $|D|$ values of 1 cm^{-1} and 0.15 cm^{-1}, respectively, and E/D values of 0.15 and 0.20, respectively.[47]

7. Local ZFS parameters

The calculated local ZFS values (Table S3) are used to deduce the global ZFS of the tetranuclear cluster for each isomer, which results from the coupling of the individual Mn ions.

Table S3. Calculated Zero Field Splitting Parameters by the multireference L-CASSCI approach for the two Mn(III) ions of the tetranuclear cluster of the three isomers of the S_1 state of the OEC.

Str.	Site	D_z	D_x	D_y	D	E/D
**S_1^A**	Mn1	-1.99	0.85	1.14	-2.99	0.05
	Mn4	-1.96	0.06	1.91	-2.95	0.31
**S_1^B**	Mn1	-2.05	0.92	1.12	-3.07	0.03
	Mn4	-2.00	0.36	1.64	-3.01	0.21
**S_1^C**	Mn1	-2.02	0.94	1.08	-3.02	0.02
	Mn4	2.30	-0.38	-1.92	3.44	0.22

In order to obtain useful expressions which can be applied to the available experimental data, three simplifying assumptions can be made. Firstly, we assume that the system is in the strong exchange limit, i.e. the isotropic interaction outweighs the local anisotropic terms, $J>>D$. For the case of multinuclear metal complexes in the strong exchange limit, it is common practice to use an effective Hamiltonian given by the giant spin approximation (GSA), which is based on the assumption that the total spin quantum number S is a good quantum number for the tetranuclear cluster. Each spin manifold of this system can be described independently, using the effective spin Hamiltonian:

$$\hat{H}_S = \mu_B B \cdot \boldsymbol{g_S} \cdot \hat{S} + \hat{S} \cdot \boldsymbol{D_S} \cdot \hat{S} \qquad \text{eq. 3}$$

where $\boldsymbol{g_s}$ describes the electronic Zeeman splitting and $\boldsymbol{D_s}$ the zero field splitting (ZFS) of the spin manifold.

The removal of the degeneracy between magnetic sublevels of the tetranuclear cluster in the absence of an applied magnetic field is described by the phenomenological spin Hamiltonian:

$$\hat{H}_{ZFS} = \hat{S} \cdot \boldsymbol{D_S} \cdot \hat{S} \qquad \text{eq. 4}$$

The axial ZFS is given from the relationship:

$$D = D_z - 1/2\,(D_x + D_y) \qquad \text{eq. 5}$$

and the rhombic anisotropy is described by:

$$\frac{E}{D} = \frac{\frac{1}{2}(D_x - D_y)}{D} \qquad \text{eq. 6}$$

The total ***D*** value of the tetranuclear cluster can be decomposed to single ion contributions from the four paramagnetic centers, Mn1(III), Mn2(IV), Mn3(IV) and Mn4(III), according to the equation:

$$\boldsymbol{D} = \sum_i d_i\,(S_i, S)\boldsymbol{D}^{(i)} + \sum_{i>j} d_{ij}\,(S_i, S_j, S)\boldsymbol{D}^{(ij)}, \quad i = 1, 2, 3, 4 \qquad \text{eq. 7}$$

where $\boldsymbol{D}^{(i)}$ is the local zero field splitting tensor of ion *i*, $D^{(ij)}$ is the dipolar ion−ion interactions between ions *i* and *j* and d_i and d_{ij} are projection coefficients. The fine-structure Mn(IV) values and spin–spin dipolar interactions are expected to be one order of magnitude smaller than the onsite Mn(III) ZFS values, so the second assumption we make is that cluster ZFS is entirely single-ion in origin:

$$\boldsymbol{D} \approx d^{(1)}\,\boldsymbol{D}^{(1)} + d^{(4)}\,\boldsymbol{D}^{(4)} \qquad \text{eq. 8}$$

Projection coefficients $d^{(i)}$ are calculated based on standard techniques[48] and depend on the individual spins of ions *i* = Mn1, Mn2, Mn3 and Mn4, on the coupling scheme and on the total spin of the cluster. Therefore, the magnitude and sign of the cluster ZFS, *D*, depends on the sign of the single-ion ZFS values, the alignment of the single-ion magnetic axes relative to each other and to the cluster axes and the magnitude and sign of the projection coefficients. Herein, we have attempted to apply those relationships to the tetranuclear cluster by successive coupling of spins to generate the global cluster spin. Based on the pairwise exchange coupling constants of the $\mathbf{S_1^A}$ structure, a natural choice of coupling scheme (third assumption) for the first magnetically excited *S* = 1 state is given by coupling S_1 and S_4 (Mn1 and Mn4) ferromagnetically, then coupling S_2 and S_3 (Mn2 and Mn3) ferromagnetically and finally coupling S_{14} and S_{23} antiferromagnetically to give the total spin *S* = 1:

$$\boldsymbol{D}_{14} = d_1\,\boldsymbol{D}^{(1)} + d_4\,\boldsymbol{D}^{(4)} \qquad \text{eq. 9}$$

$$\boldsymbol{D}_{23} = d_2\,\boldsymbol{D}^{(2)} + d_3\,\boldsymbol{D}^{(3)} \qquad \text{eq. 10}$$

$$\begin{aligned}\boldsymbol{D} = \boldsymbol{D}_{1423} &= d_{14}\,\boldsymbol{D}_{14} + d_{23}\,\boldsymbol{D}_{23} \\ &= d_{14}\left(d_1\,\boldsymbol{D}^{(1)} + d_4\,\boldsymbol{D}^{(4)}\right) + d_{23}\left(d_2\,\boldsymbol{D}^{(2)} + d_3\,\boldsymbol{D}^{(3)}\right) \\ &\approx d_{14}d_1\boldsymbol{D}^{(1)} + d_{14}d_4\boldsymbol{D}^{(4)} \end{aligned} \qquad \text{eq. 11}$$

because $D^{(2)}, D^{(3)} << D^{(1)}, D^{(4)}$

The resultant basis set for the coupling scheme: $|S_1\ S_2\ S_{12}\ S_3\ S_4\ S_{34}\ S\ M>$, or in short $|S_{12}\ S_{34}\ S\ M>$, where $|S_1 - S_2| \le S_{12} \le |S_1 + S_2|$, $|S_3 - S_4| \le S_{34} \le |S_3 + S_4|$, and $|S_{12} - S_{34}> \le S \le |S_{12} + S_{34}>$, for the subset where *S* = 1 and assuming (S_1, S_2, S_3, S_4) is (2, 3/2, 3/2, 2) is shown in the first column of Table S4. We calculated the projection coefficients, *d*, of equations 4–6 for the tetranuclear cluster using the methodology described by Bencini and Gatteschi.[48] Finally, the *D* and *E*/*D* values are calculated using

equations 5 and 6, after diagonalization of the OEC ***D*** tensor derived from equation 11. The experimental EPR spectra can be regarded as a superposition of the spectra obtained for the different total spin states.

Table S4. Spin projection coefficients and the resulting *D* (cm^{-1}) and *E*/*D* values for the tetranuclear cluster, calculated based on the chosen coupling scheme for the $\mathbf{S_1^A}$ isomer for the subset of eigenstates with *S* = 1. The calculated *D* and *E*/*D* parameters where used as initial values to simulate the *g* = 4.8 signal, giving the optimized values D_{sim} and E/D_{sim}.

Eigenstate $\lvert S_1\ S_2\ S_{12}\ S_3\ S_4\ S_{34}\ S\ M\rangle$, $S = 1$	d_1	d_4	d_2	d_3	d_{14}	d_{23}	$d_{14}\ d_1$	$d_{14}\ d_4$	$\lvert D\rvert$	E/D	D_{sim}	E/D_{sim}
|2 2 4 3/2 3/2 3 1 M>	0.21	0.21	0.2	0.2	5.5	1.5	1.18	1.18	4.62	0.18	-	-
|2 2 4 3/2 3/2 2 1 M>	0.21	0.21	0	0	28	21	6	6	23.5	0.18	-	-
|2 2 4 3/2 3/2 1 1 M>	0.21	0.21	-1.2	-1.2	49	40	10.5	10.5	41.1	0.18	-	-
|2 2 3 3/2 3/2 3 1 M>	0.1	0.1	0.2	0.2	-4.5	-4.5	-0.45	-0.45	1.76	0.18	-	-
|2 2 3 3/2 3/2 2 1 M>	0.1	0.1	0	0	3.6	0.6	0.36	0.36	1.41	0.18	0.92	0.18
|2 2 3 3/2 3/2 1 1 M>	0.1	0.1	-1.2	-1.2	15	10	1.5	1.5	5.87	0.18	-	-
|2 2 2 3/2 3/2 3 1 M>	-0.21	-0.21	0.2	0.2	0.6	3.6	-0.13	-0.13	0.5	0.18	0.15	0.23
|2 2 2 3/2 3/2 2 1 M>	-0.21	-0.21	0	0	-2.1	-2.1	0.45	0.45	1.76	0.18	-	-
|2 2 2 3/2 3/2 1 1 M>	-0.21	-0.21	-1.2	-1.2	2.1	0.1	-0.45	-0.45	1.76	0.18	-	-
|2 2 1 3/2 3/2 3 1 M>	-2.1	-2.1	0.2	0.2	10	15	-21	-21	82.2	0.18	-	-
|2 2 1 3/2 3/2 2 1 M>	-2.1	-2.1	0	0	0.1	2.1	-2.1	-2.1	8.22	0.18	-	-
|2 2 1 3/2 3/2 1 1 M>	-2.1	-2.1	-1.2	-1.2	-0.5	-0.5	1.05	1.05	4.11	0.18	-	-

For the $\mathbf{S_1^B}$ structure, the calculated exchange coupling constants indicate a ground *S* = 3 state, where S_3 is ferromagnetically coupled with S_2 and with S_4, while S_1 is antiferromagnetically coupled with S_2 and with S_4. Therefore, we adopted the coupling scheme $\lvert S_2\ S_3\ S_{23}\ S_4\ S_{234}\ S_1\ S\ M\rangle$ for the *S* = 3 ground state and the cluster's **D** tensor can be calculated using the following methodology:

$$\boldsymbol{D}_{23} = d_2\, \boldsymbol{D}^{(2)} + d_3\, \boldsymbol{D}^{(3)} \qquad \text{eq. 12}$$

$$\boldsymbol{D}_{234} = d_{23}\, \boldsymbol{D}_{23} + d_4\, \boldsymbol{D}^{(4)} \qquad \text{eq. 13}$$

$$\begin{aligned} \boldsymbol{D} = \boldsymbol{D}_{2341} &= d_{234}\, \boldsymbol{D}_{234} + d_1\, \boldsymbol{D}_1 = d_{234}\left(d_{23}\, \boldsymbol{D}_{23} + d_4\, \boldsymbol{D}^{(4)}\right) + d_1\, \boldsymbol{D}_1 \\ &= d_{234}\, d_{23}\boldsymbol{D}_{23} + d_{234}\, d_4\boldsymbol{D}^{(4)} + d_1\, \boldsymbol{D}_1 \\ &\approx d_{234}\, d_4\boldsymbol{D}^{(4)} + d_1\boldsymbol{D}^{(1)} \end{aligned} \qquad \text{eq. 14}$$

because $D_{23} << D^{(1)}, D^{(4)}$. Mn1(III) and Mn4(III) have pronounced Jahn-Teller elongation axes leading to considerably larger local ZFS values, than the Mn2(IV) and Mn3(IV) ions, which have a highly symmetric octahedral environment.

Table S5. Spin projection coefficients and the resulting *D* and *E*/*D* values for the tetranuclear cluster, calculated based on the chosen coupling scheme for the $\mathbf{S_1^B}$ isomer. The calculated *D* and *E*/*D* parameters where used as initial values to simulate the *g* ~ 12 signal, giving the optimized values D_{sim} and E/D_{sim}.

Eigenstate $\lvert S_2\ S_3\ S_{23}\ S_4\ S_{234}\ S_1\ S\ M\rangle$, $S = 3$	d_2	d_3	d_{23}	d_4	d_{234}	d_1	$d_{234}\ d_4$	d_1	$\lvert D\rvert$	E/D	D_{sim}	E/D_{sim}
|3/2 3/2 3 2 5 2 3 M>	0.2	0.2	0.33	0.13	0.17	2.17	0.02	2.17	6.69	0.03	6.77	0.05

It is important to note that there are several *D* and *E/D* combinations that reproduce the experimental signals and spectra in different frequencies could provide additional data needed to extract reliable conclusions. Given the complexity of the subject and the existence of conflicting experimental reports, reassessment of available EPR data for the S_1 and the $S_1Y_Z^{\bullet}$ states is needed.

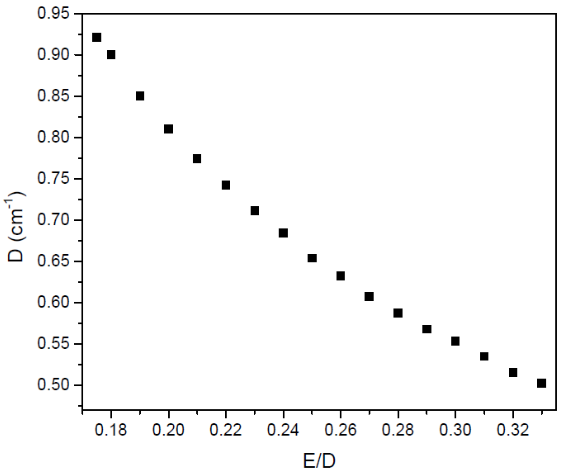

Figure S5. Various combinations of the *D* and *E*/*D* parameters of the Spin Hamiltonian for a spin state of *S* = 1 that reproduce the experimental spectrum of Figure 4b of the main text.

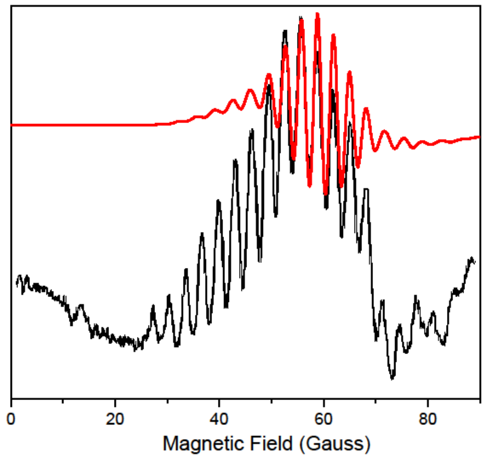

Figure S6. Simulation of the hyperfine structure (red) of the experimental multiline S_1-state EPR signal (black)[43] using an effective isotropic hyperfine coupling constant of 470 MHz for all four Mn ions in the *S* = 3 spin state.

8. Implications for the nature of the S_2 state

Although the present study focuses on the S_1 state, it is useful to discuss some aspects of the subsequent S_2 state that can be of relevance. A fundamental spectroscopic observation regarding the S_2 state of the OEC is the presence of distinct EPR spectral forms that may coexist and can be converted to each other with certain treatments, indicating that both correspond to distinct ground-state species. One of the spectral forms corresponds to a low-spin state of the cluster (S = 1/2) associated with a multiline signal at g = 2, while other signals (at $g \geq 4.1$) arise from states of higher spin multiplicity ($S \geq 5/2$), whose precise nature may differ between different organisms and PSII preparations.[49-65]

The precise origin of these signals has been the subject of various debates, but it is now almost universally accepted that the low-spin (S = 1/2) form corresponds to a structural configuration of the cluster with a Mn1-Mn2-Mn3-Mn4 oxidation state distribution of III-IV-IV-IV. This is depicted as model "$\mathbf{S_2^A}$" in Figure 1 of the main text. According to broken-symmetry DFT calculations, the exchange coupling constants for a structure of this type lead naturally to an S = 1/2 ground state.[19, 66] Moreover, ^{55}Mn hyperfine coupling constants computed with DFT-based spin-projection techniques[19, 66-67] also agree well with those obtained experimentally by electron–nucleus double resonance (ENDOR) spectroscopy[58, 64, 68] for this signal. Computed structural features, specifically Mn–Mn distances, are also in line with structural data from extended X-ray absorption fine structure (EXAFS) spectroscopy.

In contrast to the above, different ideas exist regarding the precise origin of the other type of signal of the S_2 state, the signal that corresponds to a spin $S \geq 5/2$ configuration of the cluster. Three main ideas are currently considered:

a) *Valence isomerism*: according to this scenario (historically the first explanation of the spectroscopic polymorphism in the S_2 state of the OEC), the higher-spin signals are attributed to an alternative oxidation state distribution of the cluster, IV-IV-IV-III (model shown as "$\mathbf{S_2^B}$" in Figure 1 of the main text).[66] Low-barrier relocation of the Mn(III) ion, facilitated by a simple shift of the central O5 bridge, inverts the J_{12} exchange coupling from antiferromagnetic to ferromagnetic and attenuates the J_{34} antiferromagnetic coupling, leading to a ground state spin of S = 5/2 or higher.[66]

b) *Protonation of oxo bridge*: according to this scenario the high-spin form arises when the O4 oxo bridge of the S = 1/2 conformation is protonated to OH.[69] This weakens the J_{34} antiferromagnetic exchange coupling (while Mn oxidation states remain III-IV-IV-IV) so that a higher spin state can become the ground state of the protonated form.

c) *Water coordination*: according to this scenario a high-spin form of the S_2 state can arise when a water molecule coordinates internally as an OH group to the Mn1 ion of the "normal" S = 1/2 form.[70]

It is important to stress that the above options are *not* mutually exclusive in principle. In contrast to the low-spin form, there is a paucity of fine-grained spectroscopic data on the (minority) high-spin form –or, more correctly, forms– to allow precise electronic structure correlations. The total spin state ($S \geq 5/2$) is a necessary but not sufficient criterion to judge the fitness of a computational model. Indeed, we see no compelling reason, on experimental grounds, to exclude the possibility that different high-spin forms arise under different conditions/treatments, and that these different high-spin forms may be rationalized using different scenarios.

Nevertheless, it is still possible to outline a few major arguments that favor certain possibilities over the others. First, EPR studies of the high-spin state of S_2 place the z-axis of the ZFS tensor of Mn(III) explicitly on the Mn4 ion and along Mn4–W1 vector.[65] Only the valence isomerism scenario accommodates this fact, whereas the other hypotheses do not. Second, experimental facts require that the two forms that give rise to the two signals are very close in energy because they can coexist at very low temperatures.[57,

[71-72] Experimental estimates place the energy difference at less than 1 kcal mol^{-1}.[73] The valence isomerism scenario accommodates this fact perfectly, because all existing independent computational studies place the valence isomer $\mathbf{S_2^B}$ within 2 kcal mol^{-1} of $\mathbf{S_2^A}$: for example, ca. 1 kcal mol^{-1} according to Pantazis et al.[19, 66] and Siegbahn,[74] 1.1 kcal mol^{-1} according to Bovi et al.,[75] Narzi et al.,[76] Ugur et al.,[77] and Boussac et al.,[78] 0.8 kcal mol^{-1} according to Vinyard et al.[73] and 1.3 kcal mol^{-1} according to Isobe et al.[79] Coupled-cluster calculations suggested an energy difference of 2 kcal mol^{-1} according to Saitow et al.[80] and about 1 kcal mol^{-1} according to Miyagawa et al.[81] Therefore, there is no dispute about the energetics of the valence isomeric forms $\mathbf{S_2^A}$/$\mathbf{S_2^B}$ and their perfect agreement with experiment. In stark contrast, the scenario of O4 protonation is strongly disfavored energetically because the chemically unlikely protonation of the oxo bridge comes with a heavy energetic penalty of at least 9 kcal mol^{-1} according to Corry and O'Malley.[69] In view of the above, the scenario of O4 protonation is severely disadvantaged because of the unfavorable energetics and because it disagrees with the EPR (location of Mn(III) ion) on the high-spin form of S_2, therefore it is overall the least supported scenario, theoretically and experimentally, and the least likely to provide a realistic model of the high-spin S_2 state of the OEC. The energetics pertaining to water binding are more difficult to evaluate with confidence because H_2O/OH binding is by definition a multi-step process and does not produce isomeric forms. Finally, we note that it is inadvisable to place much weight on arguments that either favor or disfavor any computational model on the basis of agreement with selected structural data, or worse, on the basis of *absence* of data.[82] Such arguments can be misleading because they assume a level of accuracy and precision that is outside the achievable information content of experimental structural studies, particularly with respect to resolving coexisting isomers/states.

We stress that the present work on the electronic structure and spectroscopic properties of the S_1 state, the orientational Jahn–Teller isomerism, the zero-field splitting analysis of Jahn–Teller isomers $\mathbf{S_1^A}$/$\mathbf{S_1^B}$, and the interpretations of EPR data on the S_1 state are entirely independent of the validity of any scenario regarding the S_2 state. No assumption about the electronic structure of the S_2 state is necessary and no knowledge of the S_2 state is required for studying the S_1 state. However, a connection does arise naturally when we study the *oxidation products* of the S_1 isomers. As discussed in the main text, oxidation of $\mathbf{S_1^A}$ and $\mathbf{S_1^B}$ is localized on *different* Mn ions (Mn1 and Mn4, respectively), which leads automatically and without any previous assumptions to the two valence isomeric forms of S_2, i.e. $\mathbf{S_2^A}$ (III-IV-IV-IV) and $\mathbf{S_2^B}$ (IV-IV-IV-III).

Thus, valence isomerism as a scenario for the S_2 state follows as a consequence of the orientational Jahn–Teller isomerism in the S_1 state. In other words, if the idea of valence isomerism in the S_2 state had never been conceived prior to the present study, it would emerge as an inescapable *outcome* of the present study.

9. References

[1] F. Neese, F. Wennmohs, U. Becker, C. Riplinger, *J. Chem. Phys.* **2020**, *152*, 224108.
[2] A. Tanaka, Y. Fukushima, N. Kamiya, *J. Am. Chem. Soc.* **2017**, *139*, 1718-1721.
[3] J. P. Perdew, *Phys. Rev. B* **1986**, *33*, 8822-8824.
[4] A. D. Becke, *Phys. Rev. A* **1988**, *38*, 3098-3100.
[5] F. Weigend, R. Ahlrichs, *Phys. Chem. Chem. Phys.* **2005**, *7*, 3297-3305.
[6] A. Schäfer, C. Huber, R. Ahlrichs, *J. Chem. Phys.* **1994**, *100*, 5829-5835.
[7] D. A. Pantazis, X. Y. Chen, C. R. Landis, F. Neese, *J. Chem. Theory Comput.* **2008**, *4*, 908-919.
[8] V. Barone, M. Cossi, *J. Phys. Chem. A* **1998**, *102*, 1995-2001.
[9] V. N. Staroverov, G. E. Scuseria, J. Tao, J. P. Perdew, *J. Chem. Phys.* **2003**, *119*, 12129-12137.
[10] F. Neese, F. Wennmohs, A. Hansen, U. Becker, *Chem. Phys.* **2009**, *356*, 98-109.
[11] K. K. Stavrev, M. C. Zerner, *Int. J. Quantum Chem.* **1997**, *65*, 877-884.
[12] J. M. Foster, S. F. Boys, *Rev. Mod. Phys.* **1960**, *32*, 300-302.
[13] M. Retegan, N. Cox, D. A. Pantazis, F. Neese, *Inorg. Chem.* **2014**, *53*, 11785-11793.
[14] S. Stoll, A. Schweiger, *J. Magn. Reson.* **2006**, *178*, 42-55.
[15] I. B. Bersuker, in *Electronic Structure and Properties of Transition Metal Compounds*, John Wiley & Sons, Hoboken, **2010**, pp. 324-391.
[16] S. Romain, C. Duboc, F. Neese, E. Rivière, L. R. Hanton, A. G. Blackman, C. Philouze, J.-C. Leprêtre, A. Deronzier, M.-N. Collomb, *Chem. Eur. J.* **2009**, *15*, 980-988.
[17] Q. Scheifele, C. Riplinger, F. Neese, H. Weihe, A.-L. Barra, F. Juranyi, A. Podlesnyak, P. L. W. Tregenna-Piggott, *Inorg. Chem.* **2008**, *47*, 439-447.
[18] P. E. M. Siegbahn, *Biochim. Biophys. Acta Bioenerg.* **2013**, *1827*, 1003-1019.
[19] V. Krewald, M. Retegan, N. Cox, J. Messinger, W. Lubitz, S. DeBeer, F. Neese, D. A. Pantazis, *Chem. Sci.* **2015**, *6*, 1676-1695.
[20] M. Suga, F. Akita, K. Hirata, G. Ueno, H. Murakami, Y. Nakajima, T. Shimizu, K. Yamashita, M. Yamamoto, H. Ago, J.-R. Shen, *Nature* **2015**, *517*, 99-103.
[21] S. Luber, I. Rivalta, Y. Umena, K. Kawakami, J. R. Shen, N. Kamiya, G. W. Brudvig, V. S. Batista, *Biochemistry* **2011**, *50*, 6308-6311.
[22] M. Shoji, H. Isobe, S. Yamanaka, M. Suga, F. Akita, J.-R. Shen, K. Yamaguchi, *Chem. Phys. Lett.* **2015**, *627*, 44-52.
[23] K. M. Davis, Y. Pushkar, *J. Phys. Chem. B* **2015**, *119*, 3492-3498.
[24] J. Yano, V. Yachandra, *Chem. Rev.* **2014**, *114*, 4175-4205.
[25] C. Glöckner, J. Kern, M. Broser, A. Zouni, V. Yachandra, J. Yano, *J. Biol. Chem.* **2013**, *288*, 22607-22620.
[26] A. Grundmeier, H. Dau, *Biochim. Biophys. Acta Bioenerg.* **2012**, *1817*, 88-105.
[27] P. Chernev, I. Zaharieva, E. Rossini, A. Galstyan, H. Dau, E.-W. Knapp, *J. Phys. Chem. B* **2016**, *120*, 10899-10922.
[28] H. Dau, A. Grundmeier, P. Loja, M. Haumann, *Phil. Trans. R. Soc. B* **2008**, *363*, 1237-1243.
[29] M. Shoji, H. Isobe, S. Yamanaka, Y. Umena, K. Kawakami, N. Kamiya, J. R. Shen, T. Nakajima, K. Yamaguchi, in *Adv. Quantum Chem., Vol. 70* (Eds.: R. S. John, J. B. Erkki), Academic Press, **2015**, pp. 325-413.
[30] K. Yamaguchi, M. Shoji, H. Isobe, S. Yamanaka, Y. Umena, K. Kawakami, N. Kamiya, *Mol. Phys.* **2017**, *115*, 636-666.
[31] J. H. A. Nugent, I. P. Muhiuddin, M. C. W. Evans, *Biochemistry* **2002**, *41*, 4117-4126.
[32] D. Koulougliotis, J.-R. Shen, N. Ioannidis, V. Petrouleas, *Biochemistry* **2003**, *42*, 3045-3053.
[33] D. Koulougliotis, C. Teutloff, Y. Sanakis, W. Lubitz, V. Petrouleas, *Phys. Chem. Chem. Phys.* **2004**, *6*, 4859-4863.
[34] V. Petrouleas, D. Koulougliotis, N. Ioannidis, *Biochemistry* **2005**, *44*, 6723-6728.
[35] G. Sioros, D. Koulougliotis, G. Karapanagos, V. Petrouleas, *Biochemistry* **2007**, *46*, 210-217.
[36] K. G. V. Havelius, J. Sjöholm, F. Ho, F. Mamedov, S. Styring, *Appl. Magn. Reson.* **2010**, *37*, 151-176.
[37] S. L. Dexheimer, M. P. Klein, *J. Am. Chem. Soc.* **1992**, *114*, 2821-2826.
[38] D. Koulougliotis, D. J. Hirsh, G. W. Brudvig, *J. Am. Chem. Soc.* **1992**, *114*, 8322-8323.
[39] M. Kusunoki, *Photochem. Photobiol. B* **2011**, *104*, 100-110.
[40] R. Pokhrel, G. Brudvig, *Phys. Chem. Chem. Phys.* **2014**, *16*, 11812-11821.
[41] T. Yamauchi, H. Mino, T. Matsukawa, A. Kawamori, T.-a. Ono, *Biochemistry* **1997**, *36*, 7520-7526.
[42] K. A. Campbell, W. Gregor, D. P. Pham, J. M. Peloquin, R. J. Debus, R. D. Britt, *Biochemistry* **1998**, *37*, 5039-5045.
[43] K. A. Campbell, J. M. Peloquin, D. P. Pham, R. J. Debus, R. D. Britt, *J. Am. Chem. Soc.* **1998**, *120*, 447-448.
[44] M. Retegan, D. A. Pantazis, *J. Am. Chem. Soc.* **2017**, *139*, 14340-14343.
[45] M. K. Chan, W. H. Armstrong, *J. Am. Chem. Soc.* **1991**, *113*, 5055-5057.

[46] C. Zhang, C. Chen, H. Dong, J.-R. Shen, H. Dau, J. Zhao, *Science* **2015**, *348*, 690-693.
[47] S. Paul, N. Cox, D. A. Pantazis, *Inorg. Chem.* **2017**, *56*, 3875-3888.
[48] A. Bencini, D. Gatteschi, *EPR of Exchange Coupled Systems*, Springer Verlag, Berlin, **1990**, p. 287.
[49] G. C. Dismukes, Y. Siderer, *Proc. Natl. Acad. Sci. U. S. A.* **1981**, *78*, 274-278.
[50] O. Hansson, L. E. Andreasson, *Biochim. Biophys. Acta* **1982**, *679*, 261-268.
[51] G. W. Brudvig, J. L. Casey, K. Sauer, *Biochim. Biophys. Acta Bioenerg.* **1983**, *723*, 366-371.
[52] J. L. Casey, K. Sauer, *Biochim. Biophys. Acta Bioenerg.* **1984**, *767*, 21-28.
[53] J. L. Zimmermann, A. W. Rutherford, *Biochim. Biophys. Acta Bioenerg.* **1984**, *767*, 160-167.
[54] J. C. De Paula, W. F. Beck, G. W. Brudvig, *J. Am. Chem. Soc.* **1986**, *108*, 4002-4009.
[55] J. L. Zimmermann, A. W. Rutherford, *Biochemistry* **1986**, *25*, 4609-4615.
[56] O. Hansson, R. Aasa, T. Vanngard, *Biophys. J.* **1987**, *51*, 825-832.
[57] A. Boussac, J.-J. Girerd, A. W. Rutherford, *Biochemistry* **1996**, *35*, 6984-6989.
[58] J. M. Peloquin, K. A. Campbell, D. W. Randall, M. A. Evanchik, V. L. Pecoraro, W. H. Armstrong, R. D. Britt, *J. Am. Chem. Soc.* **2000**, *122*, 10926-10942.
[59] J. M. Peloquin, R. D. Britt, *Biochim. Biophys. Acta Bioenerg.* **2001**, *1503*, 96-111.
[60] A. Haddy, K. V. Lakshmi, G. W. Brudvig, H. A. Frank, *Biophys. J.* **2004**, *87*, 2885-2896.
[61] M.-F. Charlot, A. Boussac, G. Blondin, *Biochim. Biophys. Acta Bioenerg.* **2005**, *1708*, 120-132.
[62] A. Haddy, *Photosynth. Res.* **2007**, *92*, 357-368.
[63] L. V. Kulik, B. Epel, W. Lubitz, J. Messinger, *J. Am. Chem. Soc.* **2007**, *129*, 13421-13435.
[64] N. Cox, L. Rapatskiy, J.-H. Su, D. A. Pantazis, M. Sugiura, L. Kulik, P. Dorlet, A. W. Rutherford, F. Neese, A. Boussac, W. Lubitz, J. Messinger, *J. Am. Chem. Soc.* **2011**, *133*, 3635-3648.
[65] H. Mino, H. Nagashima, *J. Phys. Chem. B* **2020**, *124*, 128-133.
[66] D. A. Pantazis, W. Ames, N. Cox, W. Lubitz, F. Neese, *Angew. Chem., Int. Ed.* **2012**, *51*, 9935-9940.
[67] D. A. Pantazis, M. Orio, T. Petrenko, S. Zein, E. Bill, W. Lubitz, J. Messinger, F. Neese, *Chem. Eur. J.* **2009**, *15*, 5108-5123.
[68] L. V. Kulik, B. Epel, W. Lubitz, J. Messinger, *J. Am. Chem. Soc.* **2005**, *127*, 2392-2393.
[69] T. A. Corry, P. J. O'Malley, *J. Phys. Chem. Lett.* **2019**, *10*, 5226-5230.
[70] Y. Pushkar, A. K. Ravari, S. C. Jensen, M. Palenik, *J. Phys. Chem. Lett.* **2019**, *10*, 5284-5291.
[71] A. Boussac, H. Kuhl, S. Un, M. Rögner, A. W. Rutherford, *Biochemistry* **1998**, *37*, 8995-9000.
[72] A. Boussac, S. Un, O. Horner, A. W. Rutherford, *Biochemistry* **1998**, *37*, 4001-4007.
[73] D. J. Vinyard, S. Khan, M. Askerka, V. S. Batista, G. W. Brudvig, *J. Phys. Chem. B* **2017**, *121*, 1020-1025.
[74] P. E. M. Siegbahn, *Phys. Chem. Chem. Phys.* **2018**, *20*, 22926-22931.
[75] D. Bovi, D. Narzi, L. Guidoni, *Angew. Chem., Int. Ed.* **2013**, *52*, 11744-11749.
[76] D. Narzi, D. Bovi, L. Guidoni, *Proc. Natl. Acad. Sci. U. S. A.* **2014**, *111*, 8723-8728.
[77] I. Ugur, A. W. Rutherford, V. R. I. Kaila, *Biochim. Biophys. Acta Bioenerg.* **2016**, *1857*, 740-748.
[78] A. Boussac, I. Ugur, A. Marion, M. Sugiura, V. R. I. Kaila, A. W. Rutherford, *Biochim. Biophys. Acta Bioenerg.* **2018**, *1859*, 342-356.
[79] H. Isobe, M. Shoji, J.-R. Shen, K. Yamaguchi, *J. Phys. Chem. B* **2015**, *119*, 13922-13933.
[80] M. Saitow, U. Becker, C. Riplinger, E. F. Valeev, F. Neese, *J. Chem. Phys.* **2017**, *146*, 164105.
[81] K. Miyagawa, T. Kawakami, Y. Suzuki, H. Isobe, M. Shoji, S. Yamanaka, M. Okumura, T. Nakajima, K. Yamaguchi, *J. Photochem. Photobiol., A* **2021**, *405*, 112923.
[82] R. Chatterjee, L. Lassalle, S. Gul, F. D. Fuller, I. D. Young, M. Ibrahim, C. de Lichtenberg, M. H. Cheah, A. Zouni, J. Messinger, V. K. Yachandra, J. Kern, J. Yano, *Physiol. Plant.* **2019**, *166*, 60-72.

10. Cartesian coordinates of the presented models

S_1^A

Mn	24.870550614849	35.433112514647	60.799044227707
Mn	27.294521836946	35.161927521964	62.093940680930
Mn	27.272227525506	33.229958036570	60.063924395815
Mn	27.486468677085	33.172467040968	57.318879837672
Ca	27.782726409250	36.515180245048	58.982214966436
O	26.398107262354	36.440849667309	61.080393634024
O	28.318448797415	34.630342299462	60.715281473681
O	25.946855241245	33.984400149420	61.442440076425
O	28.486780465743	32.622839908198	58.856512906653
O	26.611766011144	34.213406800743	58.701196070303
O	26.366528510644	33.808222262816	55.950985271937
O	28.337594568326	31.728719520206	55.993955431336
O	27.494940031219	36.977885276419	56.556090971088
O	28.270822797331	38.888083689943	58.752840382858
H	30.386279035995	26.554955924804	57.103004998242
C	30.312281916900	27.064948174836	58.050496991042
H	30.925319038249	26.579769914316	58.850733006886
C	28.841257653680	27.285416871320	58.415284304080
C	28.123879857697	28.161794423213	57.376143078861
O	28.747445478909	29.198276358090	56.936397667008
O	26.955937541792	27.823718203391	57.005100268925
H	33.765458970866	42.205889037510	52.647136022140
C	33.048045065308	41.804449930424	53.345645963674
H	33.479305973306	40.971406024274	53.939090006055
C	31.749224692971	41.424785919484	52.607924699491
C	30.605300357251	41.179226640918	53.566575476921
C	29.967460202853	42.256314313553	54.219155762166
C	30.201048409713	39.873232258623	53.905814476381
C	28.988701915922	42.042552214818	55.201519161819
C	29.218900465900	39.639175778737	54.879601594481
C	28.621562700451	40.722036377842	55.555013670287
O	27.711773531026	40.460113524807	56.550388878481
H	33.875561007353	32.213249988017	54.592799992651
C	32.897070989156	31.686966017370	54.550602008467
C	31.707491200654	32.621332820866	54.485646187251
O	30.551186599274	32.189626966157	54.286472834422
C	32.752121749958	30.704143987723	55.733847855110
O	32.709473435897	31.389748083654	56.991846007060
N	31.986927717394	33.943011314296	54.648590980515
C	30.995499051041	35.003102716496	54.540826476612
C	31.179878288675	35.859798280522	53.269528332612
O	30.506106938824	36.891001733437	53.104091281492
C	30.883707561759	35.837548507210	55.822341838467
C	29.934453732567	35.253694591983	56.877511067605
O	29.137712573059	34.337562823435	56.514488947643
O	29.958853239864	35.781516334002	58.046521435905
N	32.051155243850	35.394528193486	52.336578525743
C	32.064646995014	35.924467996743	50.969467993630
C	30.860047014798	35.427152998771	50.158323000353
H	21.689022998075	40.969416996549	58.988049991891
C	22.547088003069	41.319581010937	59.569561008110
C	22.885704903322	42.783792829198	59.455859061545
O	23.647287490902	43.325925227854	60.295786784587
C	23.733849000813	40.375974730543	59.232114490582
C	23.186129005634	38.941165726672	59.068315740945
C	24.215780063006	37.824631642825	59.205969607668
O	25.265526231594	37.783415466090	58.515505251417
O	23.879687175095	36.922533154848	60.082021046708
N	22.363002615862	43.500331716672	58.431657537126
C	22.797167002298	44.863986001662	58.165327996141
H	21.965681995721	45.497421999893	57.900025004254
C	23.809561519302	44.961499734367	56.991511806942
C	25.052217968762	44.165123426149	57.207888502850
C	25.380820100671	42.876709306179	56.820443205031
N	26.106020349428	44.588468835358	58.001912845642
C	27.011787539873	43.588265366770	58.083092339866
N	26.596220511653	42.534320338008	57.378091159678
H	28.443634996960	49.488006997619	59.951731998808
C	27.906209004592	50.447117003853	60.002687999243
C	26.716623470806	50.311312906091	59.088007621329
O	26.818410136658	50.324105887083	57.848055076235
H	28.589936088110	51.232480083892	59.638795026848
N	25.535123045616	50.086288928515	59.733379408697
C	24.423093995593	49.401851006928	59.075556003691
C	23.290712001747	50.340170001823	58.685933998244
C	24.027696369309	48.203589427167	59.951602829199
C	25.172633500805	47.185390225852	60.008346352157
N	25.308526882444	46.447101325678	61.118881313586
O	25.938682888087	47.053519337856	59.016702130006
H	19.541687302453	31.121287725144	59.936987021964
C	20.341428968784	30.748636818916	59.273232651006
C	21.351383021644	30.005885373748	60.144906012385
O	21.538737290453	30.318324231501	61.342455193835
C	20.992669185472	31.927529478941	58.493922229141
C	21.725716628542	32.918908424540	59.346750472405
C	23.045666718075	33.341078279427	59.340584767455
N	21.129717251719	33.632057614558	60.380586192887
C	22.054200356206	34.431209420208	60.962754144460
N	23.233340454255	34.274309786762	60.350402261788
N	22.054356867441	29.021361582344	59.527691163043
C	23.233316585270	28.373878089527	60.116344703132
H	22.974032822582	28.033225344892	61.134841714982
C	24.462325728123	29.302576549275	60.169849398608
C	24.853726729795	29.850996951373	58.795773602182
C	25.764383820503	31.074995438663	58.760082892957
O	26.053340992549	31.656978176680	59.878568418990
O	26.150652322478	31.460854149683	57.623888267987
H	25.342343007705	28.901557000619	65.956375982850
C	25.430582980449	29.593703973958	66.782123043760
H	25.235584008913	29.098263016923	67.755772979535
C	24.432222185628	30.767593581736	66.620581556389
C	24.406355289737	31.362796883698	65.244166649777
C	25.204970582303	32.301271928191	64.613907846822
N	23.492784982576	30.961574796294	64.274251167601
C	23.714447025726	31.613437405112	63.116833663460
N	24.757025042796	32.429810707763	63.309262685290
H	24.889820024761	37.055718992135	67.047361955810
C	25.452203968890	36.885366016493	66.121681047167
C	26.288291829673	38.161895772646	65.920592849171

O	25.808093857467	39.203010153257	65.429349097142
C	24.417123475439	36.616191367614	65.028642790083
C	24.954667028710	36.000343941186	63.745747720953
O	26.198087526878	35.683390070599	63.724512726455
O	24.130905499547	35.826972060931	62.791562369777
N	27.578289961990	38.128346634077	66.378296060952
C	28.490867014922	39.280093994839	66.368274015273
C	29.457222496892	39.369460253400	65.169702762791
O	30.201242212342	40.358436102505	65.046183339502
H	29.083428992064	39.257539000459	67.291200983430
N	29.416421288688	38.320283397139	64.320468859431
C	30.086844374879	38.254084828868	63.022330518544
C	29.289122852379	37.280627577334	62.125417062909
O	29.333762332212	37.383489943148	60.888049623262
C	31.561212126476	37.820169071086	63.134442686651
O	28.616448663422	36.386043706309	62.800595513502
O	24.281904842763	42.741479513764	62.778240769934
O	25.386961714585	39.097467970394	56.069868690087
O	24.822380040901	31.823701793156	54.801258877735
O	30.378006311717	30.717757079394	58.354755655104
O	26.328158555318	39.270451272405	61.779480973841
O	24.174648880167	40.012617693107	63.336901848355
O	25.581691877314	37.745644926447	53.705538975486
O	26.771224521854	43.911159293462	61.967422806029
O	28.063995888173	41.464335055979	61.652189522961
O	27.257836684482	35.605720770544	54.293966818093
H	30.699471978736	30.376336963709	66.032443930364
C	31.018906047248	30.890758078480	65.104316160892
H	31.997505979473	31.360957972776	65.227863930519
C	30.077814116134	31.996108172214	64.582839138275
C	28.824681154645	31.484791933758	63.842390254873
C	28.243603443157	32.499428018528	62.862004193562
O	28.024295601732	33.692249375357	63.288162558663
O	27.997916144748	32.099591411332	61.673460302185
H	36.469286994116	31.575153988420	63.532723982091
C	36.987893017548	32.139355031265	62.737355049932
H	37.705290989773	32.836250980235	63.183208969243
C	35.985078452978	32.867664592803	61.829308892853
C	35.167090922243	33.940665801604	62.567111809039
C	34.133662222564	34.670807220732	61.700890419728
N	33.040856983696	33.770530030693	61.310263744341
C	32.056162164704	34.077157368159	60.436075636366
N	32.022151203314	35.279486523256	59.840978305100
N	31.138598422890	33.148798805668	60.122175101101
O	23.991894960610	45.671522942584	63.479742029574
O	29.238868991681	29.787564990134	60.815741968450
O	25.986774995108	29.258848169231	55.023141069397
H	30.794807437946	28.046996234570	57.895185309259
H	28.291627424907	26.332110114741	58.509225539948
H	28.759454009770	27.811804436500	59.387539599959
H	32.836498675699	42.617741036027	54.066768397706
H	31.914955774892	40.528591841059	51.980562441622
H	31.486056452387	42.249676894013	51.916206793357
H	30.247438133222	43.288692066389	53.963885428449
H	30.655538470745	39.008180321258	53.408206968643
H	28.518571177371	42.897177682778	55.699764841721
H	28.912873554670	38.617868654928	55.138746643359
H	27.271596478049	41.401846145583	56.968812548330
H	32.896946660841	31.093493238406	53.615255973069
H	33.615483905090	30.006842209841	55.717812423972
H	31.835320739857	30.101075347399	55.585852227034
H	31.850627278210	31.160140398042	57.450738792022
H	32.944949285824	34.199801084649	54.890108861751
H	30.022414708756	34.493064153853	54.396125324502
H	30.468322859259	36.829757576476	55.562829790843
H	31.874156683330	36.021759264834	56.278465795415
H	32.483046452349	34.486366088185	52.504344555981
H	32.062006472800	37.026914714512	51.031372947735
H	33.016539595172	35.616296325090	50.503551137392
H	29.913593548096	35.725255076539	50.640662416671
H	30.879121000441	34.326398003105	50.066667000359
H	30.876869295883	35.855409691070	49.139403556616
H	22.270380324941	41.147860839652	60.628089217217
H	24.491185423843	40.414119652363	60.035445450493
H	24.233213828906	40.698944013733	58.301271354426
H	22.712516066288	38.837165328171	58.071497910542
H	22.398270774655	38.752314732462	59.817702431102
H	21.798315218689	43.013772771038	57.735251592713
H	23.246820594086	45.240545908367	59.097129494368
H	23.319841471123	44.609977265208	56.064749548025
H	24.048780876604	46.030984872515	56.840566164661
H	24.831962778857	42.185662894018	56.177638636346
H	26.173336125044	45.533784385714	58.463410213438
H	27.945980958549	43.654337284554	58.642465682183
H	27.639646809426	50.647956433064	61.053551252592
H	25.557690350772	50.068818411013	60.752872416687
H	24.853532023804	48.992163960960	58.143342689560
H	22.816477135634	50.807722854069	59.569735087219
H	22.507389325718	49.795455431820	58.127786427092
H	23.675956650754	51.145547215493	58.037324199339
H	23.151819610152	47.693493587253	59.505231350970
H	23.723557952221	48.520782346729	60.967774855745
H	25.984916089534	45.666734768662	61.155504254833
H	24.638403120411	46.484999978600	61.897319409980
H	19.872681562875	30.065735595767	58.541290817764
H	20.195293125633	32.440202813904	57.920939423674
H	21.707827331996	31.535092786119	57.749227372516
H	23.859942933433	33.053523375298	58.674037619590
H	21.855103589129	35.100163783890	61.800557640495
H	21.864843233214	28.851147908948	58.540587743690
H	23.443267282248	27.477383345114	59.507198004070
H	24.247223307092	30.138763055336	60.855670497628
H	25.304696155933	28.742424177597	60.617475596402
H	25.341877920619	29.076587665189	58.173373941619
H	23.949575882936	30.161933122022	58.234714727865
H	26.469632898840	29.968196197115	66.791291041448
H	23.409621476710	30.422369687823	66.863411375365
H	24.669645511789	31.565563965517	67.347037569764
H	26.046294911616	32.884857720630	64.987428487485
H	22.748970566300	30.274593666672	64.407374832776
H	23.118758981495	31.476272084747	62.209416697263
H	26.103543283544	36.010948407901	66.287255429212
H	23.637832109892	35.931117457910	65.412745075301
H	23.887249635347	37.546890529296	64.753500007428
H	27.910391488425	37.262435434501	66.803154094851
H	27.890012782826	40.205771956274	66.367451337477
H	28.733562577022	37.577100323146	64.490151770514
H	30.035650649128	39.250538778028	62.545436040754
H	31.640540815359	36.814513050414	63.583485761858
H	32.111915307557	38.537167805541	63.766412605666

H	32.030941235211	37.805191680498	62.135905622774
H	23.990454128045	42.844328267255	61.814563861691
H	23.852070413607	43.504865960497	63.221966336452
H	26.215179569576	39.642570682207	56.186833662451
H	25.235042379326	38.691004078346	56.968432536129
H	28.121575044323	39.456756181114	57.951443128187
H	25.241032512443	30.918917875218	54.874325872490
H	24.287487988446	31.797559102959	53.989852099057
H	29.706929596042	31.458135955573	58.460063917075
H	29.850823613534	30.039686536617	57.815424164058
H	29.165710581640	31.942926476067	55.492923473555
H	25.494079099137	39.455315560056	62.299795316668
H	26.320396930605	38.303617168640	61.568238805613
H	24.195370557972	41.005147864657	63.263553535033
H	24.649643244287	39.793985971097	64.177929661140
H	25.265857970442	38.370791672230	54.406018455263
H	25.176703091068	37.945529056178	52.836799016999
H	25.937104294914	43.507352464621	62.319854115797
H	27.315453673109	43.099589263551	61.763849130288
H	26.839877782868	37.672460651755	56.322044474593
H	27.476830687310	40.659506235840	61.657961361362
H	28.832610228183	41.207374151923	61.082191030960
H	26.848098477086	34.849771101755	54.847699416505
H	26.520363037776	36.207670086015	54.020741642333
H	31.147344757420	30.106727849153	64.333168243467
H	29.784400531588	32.680649922518	65.400270355185
H	30.660080668648	32.614982464715	63.872850504505
H	29.066625009552	30.575551801225	63.265797597500
H	28.024794745543	31.210412106631	64.558046189279
H	37.572392864006	31.403023382372	62.154978588874
H	36.523128376833	33.347332719498	60.987094417131
H	35.302977227906	32.125402029492	61.369155176346
H	34.651194540981	33.493562179927	63.442083683036
H	35.850148738586	34.707472058025	62.980419489675
H	33.725829718647	35.536515988342	62.260889135339
H	34.616877470289	35.061984628463	60.783821655213
H	31.262289730292	35.506073557365	59.161620631768
H	32.548116191777	36.054364936400	60.235287255875
H	30.254191213712	33.376442506843	59.643476204039
H	31.228051595285	32.197440294231	60.470239552575
H	24.808855020000	45.371600032275	63.953061978853
H	23.385375013687	45.958511020912	64.205675991666
H	29.709315314505	30.054194556043	59.985145985757
H	28.723996712348	30.593321426790	61.065050250663
H	26.384482955255	28.758125658264	55.816582468526
H	26.240777989779	28.714293923920	54.237005945829
H	28.949782156046	39.355189887064	59.303270070272
H	25.839045806763	33.072343290470	55.538690748206
H	28.378631341392	30.764663471199	56.271407800617
H	32.924777400489	32.913499694457	61.848935478043
H	27.520384663458	36.407757957984	55.713195786681
H	25.202481350722	33.047419051987	62.554478957429
H	20.157493528241	33.555282864879	60.679145658347
H	31.739617809370	38.519167142592	59.143009221152
H	30.532382125738	39.853502564190	57.354701037258
H	31.915330539508	40.957638808521	57.242558907600
H	31.446381486244	42.830511294570	58.268311026220
H	30.543777500394	42.977772274668	59.752974797187
H	33.169909009758	39.566185000873	58.953844008386
C	32.279197971650	39.175110014496	58.438896983249
C	31.373818495574	40.283968446056	57.929638374158
C	30.778675050901	41.068386975803	59.079418052462
N	30.928527980982	42.381353012973	59.019791969976
O	30.145591795310	40.510126733188	60.014873606930
H	32.628713348746	38.546240419538	57.601313789016

S_1^B

Mn	25.030679000000	35.351786000000	60.619164000000
Mn	27.330204000000	35.210534000000	62.133990000000
Mn	27.275849000000	33.330231000000	60.087544000000
Mn	27.475701000000	33.206302000000	57.227068000000
Ca	27.784263000000	36.581423000000	59.020207000000
O	26.444245000000	36.471877000000	61.114052000000
O	28.355036000000	34.693613000000	60.729655000000
O	26.017540000000	33.981293000000	61.532514000000
O	28.440408000000	32.765726000000	58.795554000000
O	26.375987000000	34.428355000000	58.980034000000
O	26.333948000000	33.847902000000	55.891405000000
O	28.343170000000	31.636466000000	55.834657000000
O	27.485933000000	36.984069000000	56.569078000000
O	28.327762000000	38.913445000000	58.736981000000
H	30.386279000000	26.554956000000	57.103005000000
C	30.312282000000	27.064948000000	58.050497000000
H	30.925319000000	26.579770000000	58.850733000000
C	28.837153000000	27.266803000000	58.414505000000
C	28.112088000000	28.132755000000	57.372690000000
O	28.727074000000	29.173051000000	56.932628000000
O	26.946938000000	27.786980000000	56.997786000000
H	33.765459000000	42.205889000000	52.647136000000
C	33.048045000000	41.804450000000	53.345646000000
H	33.479306000000	40.971406000000	53.939090000000
C	31.752678000000	41.422902000000	52.602910000000
C	30.606646000000	41.180405000000	53.558874000000
C	29.967992000000	42.260853000000	54.204986000000
C	30.204726000000	39.875697000000	53.905358000000
C	28.990731000000	42.051926000000	55.189510000000
C	29.223738000000	39.646589000000	54.881284000000
C	28.626415000000	40.732990000000	55.551274000000
O	27.718310000000	40.475349000000	56.548406000000
H	33.875561000000	32.213250000000	54.592800000000
C	32.897071000000	31.686966000000	54.550602000000
C	31.703527000000	32.607562000000	54.432088000000
O	30.567478000000	32.166204000000	54.154366000000
C	32.729245000000	30.731205000000	55.754648000000
O	32.641931000000	31.442579000000	56.995321000000
N	31.948807000000	33.928498000000	54.645922000000
C	30.939966000000	34.969432000000	54.518597000000
C	31.153984000000	35.849219000000	53.262966000000
O	30.488604000000	36.885907000000	53.101446000000
C	30.777337000000	35.788462000000	55.804550000000
C	29.824143000000	35.205997000000	56.853551000000
O	28.978445000000	34.333076000000	56.457526000000
O	29.874866000000	35.685719000000	58.031927000000
N	32.040067000000	35.393108000000	52.338670000000
C	32.064647000000	35.924468000000	50.969468000000
C	30.860047000000	35.427153000000	50.158323000000
H	21.689023000000	40.969417000000	58.988050000000
C	22.547088000000	41.319581000000	59.569561000000
C	22.884371000000	42.784112000000	59.455385000000

O	23.641704000000	43.328272000000	60.297796000000
C	23.737574000000	40.379385000000	59.235321000000
C	23.222254000000	38.928333000000	59.163190000000
C	24.287872000000	37.835076000000	59.204239000000
O	25.352199000000	37.903175000000	58.537062000000
O	23.966462000000	36.829111000000	59.961038000000
N	22.364260000000	43.499583000000	58.429062000000
C	22.797167000000	44.863986000000	58.165328000000
H	21.965682000000	45.497422000000	57.900025000000
C	23.810059000000	44.964804000000	56.992318000000
C	25.054432000000	44.171058000000	57.208183000000
C	25.385397000000	42.883464000000	56.820413000000
N	26.108594000000	44.597332000000	58.000257000000
C	27.017208000000	43.599965000000	58.080194000000
N	26.602682000000	42.544948000000	57.376118000000
H	28.443635000000	49.488007000000	59.951732000000
C	27.906209000000	50.447117000000	60.002688000000
C	26.716742000000	50.311254000000	59.087812000000
O	26.818823000000	50.323574000000	57.847868000000
H	28.589991000000	51.232488000000	59.638898000000
N	25.535047000000	50.086918000000	59.733022000000
C	24.423094000000	49.401851000000	59.075556000000
C	23.290712000000	50.340170000000	58.685934000000
C	24.028159000000	48.204061000000	59.952255000000
C	25.174191000000	47.187251000000	60.010327000000
N	25.307904000000	46.446823000000	61.119518000000
O	25.943162000000	47.058277000000	59.020434000000
H	19.609459000000	31.082402000000	59.903831000000
C	20.383264000000	30.739616000000	59.194838000000
C	21.431939000000	29.976553000000	60.001947000000
O	21.642083000000	30.227514000000	61.209730000000
C	20.990649000000	31.950928000000	58.430040000000
C	21.780857000000	32.898178000000	59.282306000000
C	23.093754000000	33.335170000000	59.198283000000
N	21.267598000000	33.532099000000	60.407589000000
C	22.234452000000	34.297758000000	60.967807000000
N	23.359514000000	34.195631000000	60.252862000000
N	22.142660000000	29.042917000000	59.317322000000
C	23.342961000000	28.389087000000	59.852813000000
H	23.101878000000	27.971975000000	60.847208000000
C	24.542878000000	29.348295000000	59.968215000000
C	24.926231000000	29.987378000000	58.630775000000
C	25.800144000000	31.230301000000	58.699912000000
O	26.085812000000	31.728490000000	59.843029000000
O	26.167537000000	31.717051000000	57.579186000000
H	25.342343000000	28.901557000000	65.956376000000
C	25.430583000000	29.593704000000	66.782123000000
H	25.235584000000	29.098263000000	67.755773000000
C	24.427919000000	30.763112000000	66.619093000000
C	24.412642000000	31.356767000000	65.242954000000
C	25.199720000000	32.315958000000	64.630052000000
N	23.538096000000	30.924343000000	64.250853000000
C	23.772426000000	31.579279000000	63.096843000000
N	24.783970000000	32.427427000000	63.314266000000
H	24.889820000000	37.055719000000	67.047362000000
C	25.452204000000	36.885366000000	66.121681000000
C	26.289770000000	38.161532000000	65.924151000000
O	25.804046000000	39.209693000000	65.453021000000
C	24.428769000000	36.611789000000	65.014072000000
C	24.981006000000	35.998675000000	63.727835000000
O	26.239546000000	35.703651000000	63.740897000000
O	24.186358000000	35.807520000000	62.764492000000
N	27.587133000000	38.122181000000	66.362646000000
C	28.490867000000	39.280094000000	66.368274000000
C	29.465653000000	39.398764000000	65.180175000000
O	30.184537000000	40.407665000000	65.066437000000
H	29.083429000000	39.257539000000	67.291201000000
N	29.464453000000	38.347571000000	64.334430000000
C	30.134340000000	38.297724000000	63.035797000000
C	29.339182000000	37.320275000000	62.140183000000
O	29.390095000000	37.415513000000	60.901596000000
C	31.613117000000	37.878310000000	63.141908000000
O	28.662013000000	36.435566000000	62.821770000000
O	24.282791000000	42.744689000000	62.779435000000
O	25.404384000000	39.125820000000	56.048761000000
O	24.822380000000	31.823702000000	54.801259000000
O	30.295152000000	30.761633000000	58.341690000000
O	26.342162000000	39.273285000000	61.793950000000
O	24.184132000000	40.016690000000	63.348093000000
O	25.581692000000	37.745645000000	53.705539000000
O	26.769716000000	43.912872000000	61.965024000000
O	28.067905000000	41.468023000000	61.664356000000
O	27.278043000000	35.655860000000	54.266305000000
H	30.699472000000	30.376337000000	66.032444000000
C	31.018906000000	30.890758000000	65.104316000000
H	31.997506000000	31.360958000000	65.227864000000
C	30.082907000000	32.000232000000	64.588663000000
C	28.848248000000	31.497934000000	63.814874000000
C	28.298012000000	32.533934000000	62.841338000000
O	28.101687000000	33.721943000000	63.285945000000
O	28.062492000000	32.152051000000	61.641682000000
H	36.469287000000	31.575154000000	63.532724000000
C	36.987893000000	32.139355000000	62.737355000000
H	37.705291000000	32.836251000000	63.183209000000
C	35.988665000000	32.857414000000	61.815531000000
C	35.138678000000	33.917010000000	62.537269000000
C	34.131539000000	34.656515000000	61.646502000000
N	33.048566000000	33.764264000000	61.209435000000
C	32.071800000000	34.097985000000	60.337120000000
N	32.045675000000	35.318961000000	59.775930000000
N	31.157075000000	33.182750000000	59.980690000000
O	23.991895000000	45.671523000000	63.479743000000
O	29.238869000000	29.787565000000	60.815742000000
O	25.986775000000	29.258848000000	55.023141000000
H	30.783877000000	28.052510000000	57.897939000000
H	28.300236000000	26.306341000000	58.509499000000
H	28.747588000000	27.793211000000	59.386051000000
H	32.833656000000	42.617664000000	54.065819000000
H	31.921293000000	40.525171000000	51.978522000000
H	31.492196000000	42.246124000000	51.908249000000
H	30.247049000000	43.291806000000	53.943115000000
H	30.660255000000	39.008752000000	53.411946000000
H	28.519752000000	42.908776000000	55.683191000000
H	28.917921000000	38.626789000000	55.146708000000
H	27.276493000000	41.421682000000	56.969105000000
H	32.917166000000	31.065235000000	53.634220000000
H	33.600830000000	30.044457000000	55.777789000000
H	31.824282000000	30.113637000000	55.593676000000
H	31.777720000000	31.205819000000	57.439246000000
H	32.888659000000	34.198281000000	54.938885000000

H	29.981644000000	34.446151000000	54.329832000000
H	30.343730000000	36.771304000000	55.536701000000
H	31.752795000000	35.997433000000	56.280895000000
H	32.469621000000	34.483637000000	52.505291000000
H	32.064871000000	37.026854000000	51.030994000000
H	33.017795000000	35.611888000000	50.509600000000
H	29.913801000000	35.725975000000	50.640520000000
H	30.879121000000	34.326398000000	50.066667000000
H	30.876449000000	35.854945000000	49.139142000000
H	22.271241000000	41.149829000000	60.628793000000
H	24.522219000000	40.469941000000	60.007794000000
H	24.196633000000	40.667799000000	58.272766000000
H	22.646625000000	38.783643000000	58.225970000000
H	22.520526000000	38.732378000000	59.992784000000
H	21.802683000000	43.012290000000	57.730676000000
H	23.245582000000	45.239436000000	59.098148000000
H	23.321618000000	44.613381000000	56.064848000000
H	24.047335000000	46.034897000000	56.842636000000
H	24.837362000000	42.190183000000	56.179372000000
H	26.174484000000	45.542556000000	58.462717000000
H	27.952220000000	43.668378000000	58.637865000000
H	27.639526000000	50.647931000000	61.053523000000
H	25.557547000000	50.069765000000	60.752526000000
H	24.853529000000	48.991940000000	58.143467000000
H	22.816940000000	50.808176000000	59.569738000000
H	22.507010000000	49.795427000000	58.128336000000
H	23.675898000000	51.145197000000	58.036854000000
H	23.153001000000	47.692728000000	59.505881000000
H	23.723209000000	48.521629000000	60.968069000000
H	25.985625000000	45.667563000000	61.156773000000
H	24.636358000000	46.482955000000	61.896871000000
H	19.890140000000	30.075781000000	58.461360000000
H	20.162451000000	32.489837000000	57.929263000000
H	21.658316000000	31.590658000000	57.627110000000
H	23.850336000000	33.111126000000	58.445092000000
H	22.106000000000	34.906821000000	61.863261000000
H	21.932512000000	28.912960000000	58.328413000000
H	23.573784000000	27.543283000000	59.182196000000
H	24.296180000000	30.134139000000	60.701048000000
H	25.400445000000	28.789609000000	60.387269000000
H	25.446514000000	29.262232000000	57.976066000000
H	24.020674000000	30.301981000000	58.074278000000
H	26.468483000000	29.971114000000	66.791043000000
H	23.405357000000	30.413151000000	66.855375000000
H	24.658149000000	31.561955000000	67.346752000000
H	26.015155000000	32.923559000000	65.022331000000
H	22.812006000000	30.215705000000	64.367006000000
H	23.207127000000	31.421734000000	62.173822000000
H	26.104446000000	36.012157000000	66.290191000000
H	23.649502000000	35.922826000000	65.391064000000
H	23.897539000000	37.541110000000	64.736792000000
H	27.923559000000	37.252471000000	66.776090000000
H	27.880941000000	40.199805000000	66.375682000000
H	28.788370000000	37.594691000000	64.491237000000
H	30.071659000000	39.295924000000	62.563890000000
H	31.704366000000	36.872238000000	63.587890000000
H	32.159372000000	38.598584000000	63.774169000000
H	32.080197000000	37.870563000000	62.141904000000
H	23.989951000000	42.845333000000	61.816091000000
H	23.852531000000	43.508387000000	63.222247000000
H	26.228483000000	39.680642000000	56.153688000000
H	25.249996000000	38.763674000000	56.964998000000
H	28.151066000000	39.490479000000	57.947056000000
H	25.254062000000	30.924958000000	54.866442000000
H	24.287488000000	31.797559000000	53.989852000000
H	29.636001000000	31.511084000000	58.442719000000
H	29.771139000000	30.076041000000	57.807387000000
H	29.178910000000	31.823254000000	55.340671000000
H	25.507996000000	39.458603000000	62.313487000000
H	26.340879000000	38.301981000000	61.596242000000
H	24.203027000000	41.008925000000	63.272956000000
H	24.655838000000	39.799943000000	64.191611000000
H	25.278909000000	38.379547000000	54.404560000000
H	25.176703000000	37.945529000000	52.836799000000
H	25.935704000000	43.509828000000	62.318841000000
H	27.315882000000	43.101048000000	61.767440000000
H	26.833514000000	37.686608000000	56.350845000000
H	27.483294000000	40.660619000000	61.675488000000
H	28.835074000000	41.211683000000	61.093009000000
H	26.851772000000	34.895483000000	54.794563000000
H	26.548340000000	36.267373000000	53.989461000000
H	31.146235000000	30.108387000000	64.331407000000
H	29.771642000000	32.668025000000	65.413126000000
H	30.674183000000	32.635431000000	63.901103000000
H	29.102139000000	30.595912000000	63.232202000000
H	28.029781000000	31.216666000000	64.506716000000
H	37.576652000000	31.401441000000	62.160947000000
H	36.535062000000	33.346506000000	60.984154000000
H	35.326999000000	32.105468000000	61.341297000000
H	34.596210000000	33.456716000000	63.388866000000
H	35.802981000000	34.682523000000	62.982479000000
H	33.706404000000	35.513840000000	62.206496000000
H	34.642505000000	35.061602000000	60.750733000000
H	31.277914000000	35.554557000000	59.114923000000
H	32.539188000000	36.089338000000	60.218753000000
H	30.268049000000	33.434076000000	59.520517000000
H	31.239642000000	32.220471000000	60.300323000000
H	24.808855000000	45.371600000000	63.953062000000
H	23.385375000000	45.958511000000	64.205676000000
H	29.696626000000	30.045389000000	59.974873000000
H	28.747858000000	30.609602000000	61.063878000000
H	26.381092000000	28.746045000000	55.809749000000
H	26.240778000000	28.714294000000	54.237006000000
H	28.995315000000	39.389728000000	59.295506000000
H	25.788465000000	33.114027000000	55.495283000000
H	28.382513000000	30.686063000000	56.145411000000
H	32.927843000000	32.890209000000	61.718930000000
H	27.513424000000	36.435319000000	55.713130000000
H	25.240870000000	33.063275000000	62.574371000000
H	20.324478000000	33.422345000000	60.779542000000
H	31.739276000000	38.518729000000	59.142326000000
H	30.533748000000	39.853919000000	57.352814000000
H	31.915937000000	40.958793000000	57.243775000000
H	31.441466000000	42.831387000000	58.265424000000
H	30.544495000000	42.977325000000	59.753738000000
H	33.169909000000	39.566185000000	58.953844000000
C	32.279198000000	39.175110000000	58.438897000000
C	31.373836000000	40.284217000000	57.929422000000
C	30.778675000000	41.068387000000	59.079418000000
N	30.928528000000	42.381353000000	59.019792000000

O	30.151530000000	40.512545000000	60.020331000000

S_1^C

Mn	24.920567543418	35.414343978253	60.732369922600
Mn	27.317471704611	35.180053050668	62.121193407551
Mn	27.287431913489	33.280474420499	60.077393793159
Mn	27.473421149147	33.209543143844	57.290105107462
Ca	27.773804127741	36.540665515508	58.996943191876
O	26.419873058822	36.452281212788	61.086162191749
O	28.347085994421	34.655559096618	60.736725501769
O	25.978283181591	33.984745708995	61.470137186453
O	28.479658960746	32.692917324034	58.830897902455
O	26.517359630432	34.310041893137	58.810826689886
O	26.354542871905	33.829336942970	55.929953389443
O	28.344616252841	31.693149829589	55.913809536821
O	27.497445952613	36.988982558456	56.557269442983
O	28.290964545881	38.897851170484	58.747461233022
H	30.386279020951	26.554956028178	57.103005003731
C	30.312281942659	27.064947949162	58.050496979057
H	30.925319021661	26.579770017972	58.850733013912
C	28.838545646867	27.273238256169	58.415257250673
C	28.115688868156	28.142978190496	57.374560662536
O	28.730089824383	29.186148469788	56.940578975673
O	26.952392648471	27.795136359730	56.996100575017
H	33.765459021414	42.205888969754	52.647135974700
C	33.048044977296	41.804450052681	53.345646057142
H	33.479305988649	40.971405990333	53.939089994112
C	31.750779308732	41.424265493271	52.605562467889
C	30.606014108400	41.180010233663	53.563049858393
C	29.966569732924	42.258583675052	54.211482932116
C	30.203907395969	39.874478081056	53.906377858184
C	28.988248827964	42.046868708156	55.194600200507
C	29.222166253760	39.642462498944	54.880933485881
C	28.623759439535	40.726980536246	55.552701312666
O	27.713985210435	40.466815389200	56.548440511348
H	33.875560994406	32.213250058778	54.592800053143
C	32.897070995463	31.686965911689	54.550601929337
C	31.707647572689	32.617417572653	54.454972766628
O	30.561147072707	32.184570069504	54.208787597366
C	32.737509634517	30.717807899675	55.743994975609
O	32.671320281088	31.415179787837	56.993950427394
N	31.972781481503	33.937860188105	54.649420702414
C	30.974762307208	34.990709615064	54.533277081388
C	31.171119082811	35.856613064769	53.267902879743
O	30.500880816710	36.890174360368	53.103917995922
C	30.841737645261	35.817935246299	55.817412634107
C	29.885350699275	35.236622539095	56.866685618217
O	29.064023285119	34.344034791407	56.485030321527
O	29.921330761276	35.740828570976	58.041649540577
N	32.047184566546	35.394313747721	52.337646192396
C	32.064647016936	35.924468011141	50.969467992115
C	30.860046964564	35.427152983372	50.158323036249
H	21.689023003429	40.969417001185	58.988049989086
C	22.547087993321	41.319581001306	59.569561012954
C	22.877103223021	42.786452842359	59.461556216655
O	23.621521279162	43.334623274093	60.312864735098
C	23.736908743462	40.381902089266	59.227801751916
C	23.199166848223	38.942202299177	59.082058590917
C	24.241171903689	37.833750822652	59.192390715551

H	32.629793000000	38.547411000000	57.600816000000

O	25.299007000154	37.831202735785	58.512884390564
O	23.906521551390	36.895727558509	60.029296883843
N	22.364883240394	43.499165707807	58.429239167846
C	22.797166991959	44.863986004670	58.165328002986
H	21.965682001216	45.497422000858	57.900024999419
C	23.810724661311	44.963676820336	56.992851121683
C	25.054144087740	44.168538018299	57.209798925815
C	25.384989377191	42.881188621769	56.820842973698
N	26.107021184221	44.592591599656	58.004744947368
C	27.014577602327	43.593987450748	58.084772530553
N	26.600801545445	42.540234643938	57.378486141664
H	28.443635000764	49.488006996782	59.951731997072
C	27.906208995947	50.447116999708	60.002688001558
C	26.716683706997	50.311350233788	59.087880099547
O	26.818668941137	50.323763879887	57.847933273096
H	28.589985244909	51.232478461380	59.638874407910
N	25.535079836105	50.086682004569	59.733152319921
C	24.423094007449	49.401850988607	59.075556000268
C	23.290711979457	50.340170015307	58.685933989436
C	24.027815498168	48.204016917905	59.952117057034
C	25.172622906602	47.185749684021	60.009517059638
N	25.304611603909	46.443776894559	61.117953257930
O	25.941848691383	47.056534694060	59.019868897967
H	19.591335697108	31.087607953442	59.897903058266
C	20.387305383748	30.734126418696	59.219226604504
C	21.412716178382	29.986101675670	60.068351225459
O	21.606877004971	30.277049524683	61.269819876199
C	21.018283796613	31.931893985109	58.452519422707
C	21.760062995178	32.910574007400	59.312617614462
C	23.074790387270	33.348453316294	59.283807661348
N	21.183715765260	33.587620963181	60.381249759014
C	22.115092364986	34.380273877979	60.962008217956
N	23.278588030449	34.254226893540	60.314724864278
N	22.121282421186	29.020523504063	59.427139643142
C	23.310067018095	28.374311443703	59.996959897473
H	23.056625292121	27.999323177778	61.004923168912
C	24.523916235729	29.319957504571	60.080262195432
C	24.912564672483	29.910604278399	58.722560352377
C	25.799029841833	31.149900004610	58.735073878583
O	26.091785102967	31.687975867284	59.867060539581
O	26.165687285975	31.592656200451	57.605792000406
H	25.342342956191	28.901556968484	65.956376054719
C	25.430583094499	29.593704113402	66.782122887759
H	25.235583966369	29.098262946046	67.755773033545
C	24.433425281018	30.767748789290	66.614921811340
C	24.417830336328	31.360539051130	65.237327752691
C	25.212205645085	32.309146501557	64.616608979884
N	23.525738441295	30.943293685134	64.254207786981
C	23.756362081904	31.596219889935	63.098718375364
N	24.783335602915	32.428702045805	63.304789973141
H	24.889819993089	37.055719039250	67.047361990530
C	25.452203983689	36.885365922465	66.121681020066
C	26.289027895935	38.161582221786	65.921605504699
O	25.806421707843	39.205717998084	65.438865129325
C	24.421995213727	36.612681160100	65.023113409324
C	24.967475791108	36.007656697258	63.735458314576
O	26.220278222770	35.710978218084	63.724518082585
O	24.156202591272	35.825557933617	62.777466875244

N	27.582380645454	38.125375299109	66.370769519802
C	28.490867014701	39.280094020907	66.368273924073
C	29.461035716908	39.383916943387	65.174497438330
O	30.192871883763	40.382647186417	65.055650266015
H	29.083429004625	39.257538984479	67.291201062300
N	29.438193091357	38.334296116731	64.326089529167
C	30.107879355769	38.276387421777	63.027399919373
C	29.312737933628	37.299188143834	62.131890623456
O	29.357867783131	37.397336885142	60.894359655289
C	31.585090664144	37.851671238137	63.136244260140
O	28.640283806105	36.407947246467	62.812815412389
O	24.277451038656	42.731082658452	62.784868926409
O	25.388647434032	39.122590099139	56.050771594153
O	24.822379996032	31.823701885547	54.801258982196
O	30.333948135512	30.735899122632	58.348437691714
O	26.333761613074	39.261701663423	61.782584391934
O	24.176011753043	40.001275213138	63.339559595613
O	25.581692089100	37.745644963356	53.705539024160
O	26.760973049035	43.902870009422	61.961237827676
O	28.064105713057	41.459137170165	61.660801959291
O	27.270806021594	35.634006990958	54.278631509227
H	30.699472012867	30.376337016547	66.032444005774
C	31.018905972140	30.890757960174	65.104315973149
H	31.997506011448	31.360958016192	65.227864013401
C	30.079866886743	31.998034415907	64.587172262994
C	28.826040095190	31.489896784960	63.846333591998
C	28.258288680768	32.510608250794	62.866714422311
O	28.032465379844	33.698915051171	63.303215500557
O	28.034067147195	32.123285356539	61.669935704234
H	36.469287013941	31.575153980579	63.532723984168
C	36.987892959093	32.139355033463	62.737355034715
H	37.705291019404	32.836250990776	63.183208987836
C	35.988147984824	32.862628131819	61.821175465026
C	35.151112690617	33.927134778962	62.550374047712
C	34.136669294450	34.666203444473	61.668510180669
N	33.053072083918	33.770939431548	61.241284967953
C	32.074300787514	34.096921861414	60.367818951612
N	32.042375514227	35.314726945302	59.802459532313
N	31.163374067568	33.174646015854	60.019281511704
O	23.991895142465	45.671523095077	63.479741894427
O	29.238869003982	29.787565016223	60.815742022639
O	25.986775078928	29.258848047050	55.023141013362
H	30.787944538604	28.050485648129	57.897066780648
H	28.297536341032	26.315075153198	58.509962877992
H	28.752382447354	27.799651517980	59.387121203774
H	32.835320670372	42.617719588250	54.066371509281
H	31.917726255602	40.527499990849	51.979341301802
H	31.488918614557	42.248625022125	51.912754538268
H	30.244760899669	43.290387076827	53.952014818314
H	30.659158002568	39.008614560805	53.410871322677
H	28.516881842159	42.902434016223	55.690091112477
H	28.917174433006	38.621928979013	55.144415134406
H	27.275914695689	41.410251579582	56.967752104639
H	32.907788538072	31.078720061635	53.624961653128
H	33.604262288437	30.024508972594	55.748243617594
H	31.826087117904	30.108681945688	55.587399002800
H	31.809743258408	31.180823459453	57.444920082623
H	32.921413864474	34.198203447747	54.921969707746
H	30.007096358660	34.475716115497	54.370531540617
H	30.422609097266	36.807963071717	55.554609800145
H	31.825618730089	36.008555545372	56.284638706754
H	32.480128849664	34.486788722258	52.506002213375
H	32.063180801175	37.026911907881	51.030962858637
H	33.016915776132	35.614431834256	50.505759857859
H	29.913596277091	35.725466075704	50.640510680725
H	30.879121009813	34.326397998792	50.066666987948
H	30.876844891985	35.855324369311	49.139346044969
H	22.272483105098	41.142866025659	60.627784887639
H	24.501780624270	40.431398586391	60.023406392912
H	24.224771954877	40.701037734668	58.289621051145
H	22.700223352939	38.830725730110	58.098183039521
H	22.431482791114	38.747048382124	59.850701743831
H	21.814464901686	43.007812017680	57.724803784267
H	23.245331850022	45.239336361719	59.098319652218
H	23.322630064260	44.612484754885	56.065084755332
H	24.048956097049	46.033540994260	56.843048421605
H	24.837631171042	42.189773780105	56.177147987051
H	26.173535697733	45.537895348173	58.466762797872
H	27.948558055776	43.661007793001	58.644373843853
H	27.639574953104	50.647935496522	61.053535406189
H	25.557686333830	50.069197426180	60.752645188122
H	24.853526128132	48.991958381238	58.143448077323
H	22.816774822171	50.807993270580	59.569747144332
H	22.507149860105	49.795435925364	58.128134000609
H	23.675898548805	51.145338017609	58.037029388333
H	23.151890757114	47.693621251743	59.506159348548
H	23.723650971384	48.521573106039	60.968175817834
H	25.980545670680	45.663012429910	61.154409034591
H	24.633039156814	46.480060613294	61.895329393460
H	19.916750186231	30.058727935530	58.481515953857
H	20.208544968809	32.449663646146	57.901838604925
H	21.724395666973	31.558235816848	57.689604514531
H	23.874767605815	33.093515989580	58.587134022514
H	21.932522952847	35.023467250105	61.823423909639
H	21.926196972815	28.865034708716	58.438736691290
H	23.533363015592	27.499700759148	59.361434563645
H	24.292009405752	30.133190571403	60.787728262491
H	25.373253872393	28.761378576194	60.516226057879
H	25.421071021739	29.160118322944	58.087347857075
H	24.007610031988	30.217721273265	58.160936350044
H	26.469820906780	29.967707714795	66.791523454161
H	23.409225440547	30.423053172342	66.851875141594
H	24.666974745686	31.566850928397	67.341314529362
H	26.040269708774	32.904538984333	65.001057649217
H	22.790340011882	30.245532862029	64.377547450352
H	23.176902123301	31.448598918563	62.182520006080
H	26.104343268482	36.011878389451	66.289245709701
H	23.647626818113	35.919560969472	65.402645273196
H	23.885174805708	37.540474439728	64.751592758397
H	27.916111605225	37.257964888710	66.791232043275
H	27.885670477626	40.202966376461	66.371582938591
H	28.758698554357	37.586439938612	64.489946879908
H	30.049449581401	39.273217842496	62.552108408141
H	31.671902938774	36.845621556177	63.583073681842
H	32.132379942583	38.570753281236	63.768868126881
H	32.053646739091	37.841809103089	62.137038510488
H	23.977823452404	42.842126925749	61.824502404293
H	23.850048339465	43.489206684900	63.239245497961
H	26.218732103278	39.665829701320	56.166003568035
H	25.235741852833	38.724750261309	56.952440528145

H	28.131148550189	39.470333417067	57.950876951282
H	25.247601780788	30.921979432806	54.870120578558
H	24.287487999512	31.797559068658	53.989852006625
H	29.669589562818	31.481969512521	58.453717830162
H	29.803416962002	30.053389293138	57.816240447887
H	29.171050976425	31.902582473829	55.411533391861
H	25.499474342733	39.445688710715	62.302410648610
H	26.333357777061	38.291161974628	61.582376494072
H	24.194790256643	40.993715479963	63.264878163820
H	24.651362515823	39.785345981288	64.181266155629
H	25.282552645284	38.383654019647	54.402616914680
H	25.176702941280	37.945529038939	52.836798975714
H	25.928983061754	43.497927530014	62.317428195172
H	27.308599943437	43.092274081940	61.762990395979
H	26.830800746034	37.677150117262	56.338930648942
H	27.479109585459	40.652359940910	61.667399628010
H	28.831972356309	41.204847462078	61.088991446383
H	26.854472282710	34.877083249940	54.819436397375
H	26.536991157509	36.239648001516	54.001246987156
H	31.146001788154	30.107978941026	64.331779343337
H	29.787718721117	32.680663059724	65.406585419572
H	30.662352441766	32.618011118549	63.878403648226
H	29.064794694729	30.578884162462	63.271357371727
H	28.022958571487	31.221732772162	64.560625886299
H	37.574903759904	31.402070709880	62.158511436316
H	36.532019482443	33.348713632107	60.986396983852
H	35.318329836809	32.115357502211	61.351102426025
H	34.616451033645	33.470564622762	63.408940638677
H	35.823319891100	34.691027389631	62.986341377601
H	33.714965741636	35.523647170385	62.230964687235
H	34.640253468542	35.070945411917	60.768357915611
H	31.277182125136	35.551095480184	59.134816709606
H	32.548042342422	36.084341834229	60.232384674170
H	30.274361212641	33.414029524308	59.552946509720
H	31.252651173933	32.215183494987	60.345140414841
H	24.808854953179	45.371599961082	63.953062114641
H	23.385374935296	45.958510951938	64.205676005159
H	29.698134776170	30.054469095735	59.978564651268
H	28.735997721500	30.598374646827	61.073239546763
H	26.379489498419	28.749202941639	55.812966370058
H	26.240777939886	28.714293997019	54.237005995077
H	28.966828146104	39.367799964997	59.300070136177
H	25.823137020941	33.088911703023	55.528417545577
H	28.397420438665	30.734706651823	56.197282412707
H	32.937752179290	32.897812218305	61.753491424697
H	27.519118335912	36.426594390846	55.709689854877
H	25.234546205363	33.056252638470	62.554852175432
H	20.221053486041	33.490387752687	60.703685358527
H	31.739300144555	38.518909384811	59.142519441704
H	30.533017660734	39.853870461866	57.353833484405
H	31.915826578563	40.958066885812	57.243042544168
H	31.444650752750	42.830682193135	58.267204539152
H	30.543745953402	42.977771509237	59.752960908380
H	33.169909013072	39.566184992490	58.953843982756
C	32.279198016071	39.175109998421	58.438897035680
C	31.373949734877	40.284109752717	57.929528196378
C	30.778674917561	41.068387009555	59.079417884804
N	30.928528045395	42.381353004313	59.019792054320
O	30.147382423749	40.510897158109	60.016534418164
H	32.629243670716	38.546722732912	57.601138182181

S_1^D

Mn	24.862888000000	35.406126000000	60.756724000000
Mn	27.277653000000	35.202412000000	62.125419000000
Mn	27.245514000000	33.305927000000	60.079321000000
Mn	27.693885000000	32.905869000000	57.105699000000
Ca	27.723214000000	36.656135000000	59.011126000000
O	26.362969000000	36.459945000000	61.130261000000
O	28.272276000000	34.700953000000	60.676425000000
O	25.971041000000	33.967739000000	61.485868000000
O	28.449985000000	32.726071000000	58.834407000000
O	26.279623000000	34.542071000000	58.938151000000
O	26.331620000000	33.727124000000	56.084797000000
O	28.454047000000	31.477603000000	55.695859000000
O	27.406302000000	36.944079000000	56.577751000000
O	28.306414000000	38.956203000000	58.710071000000
H	30.386279000000	26.554956000000	57.103005000000
C	30.312282000000	27.064948000000	58.050497000000
H	30.925319000000	26.579770000000	58.850733000000
C	28.839008000000	27.271081000000	58.409384000000
C	28.130771000000	28.131045000000	57.355760000000
O	28.784086000000	29.136722000000	56.881843000000
O	26.952252000000	27.819983000000	57.002425000000
H	33.765459000000	42.205889000000	52.647136000000
C	33.048045000000	41.804450000000	53.345646000000
H	33.479306000000	40.971406000000	53.939090000000
C	31.755233000000	41.413284000000	52.602778000000
C	30.608688000000	41.171953000000	53.558729000000
C	29.979398000000	42.255696000000	54.208773000000
C	30.194032000000	39.869812000000	53.900591000000
C	29.001466000000	42.052882000000	55.193101000000
C	29.210337000000	39.647464000000	54.876300000000
C	28.623272000000	40.736583000000	55.552783000000
O	27.715074000000	40.491260000000	56.550808000000
H	33.875561000000	32.213250000000	54.592800000000
C	32.897071000000	31.686966000000	54.550602000000
C	31.704282000000	32.600180000000	54.408628000000
O	30.579053000000	32.152197000000	54.091600000000
C	32.730263000000	30.743112000000	55.762561000000
O	32.650836000000	31.471433000000	56.994186000000
N	31.927433000000	33.920004000000	54.642700000000
C	30.904737000000	34.944839000000	54.499171000000
C	31.119870000000	35.834918000000	53.250926000000
O	30.425195000000	36.851389000000	53.083325000000
C	30.698794000000	35.752522000000	55.785937000000
C	29.786666000000	35.110548000000	56.833115000000
O	29.044758000000	34.140698000000	56.423232000000
O	29.761839000000	35.596777000000	58.001743000000
N	32.032209000000	35.401957000000	52.342725000000
C	32.064647000000	35.924468000000	50.969468000000
C	30.860047000000	35.427153000000	50.158323000000
H	21.689023000000	40.969417000000	58.988050000000
C	22.547088000000	41.319581000000	59.569561000000
C	22.898962000000	42.779873000000	59.444835000000
O	23.689343000000	43.311578000000	60.264567000000
C	23.731036000000	40.374938000000	59.238644000000
C	23.186379000000	38.938155000000	59.110525000000
C	24.225434000000	37.834088000000	59.236966000000
O	25.311374000000	37.851646000000	58.605523000000

O	23.858727000000	36.873439000000	60.037548000000
N	22.361914000000	43.501307000000	58.432600000000
C	22.797167000000	44.863986000000	58.165328000000
H	21.965682000000	45.497422000000	57.900025000000
C	23.803942000000	44.964436000000	56.987264000000
C	25.048931000000	44.171303000000	57.197499000000
C	25.380760000000	42.887764000000	56.799175000000
N	26.102118000000	44.594316000000	57.992604000000
C	27.013080000000	43.600932000000	58.067275000000
N	26.598253000000	42.550784000000	57.354681000000
H	28.443635000000	49.488007000000	59.951732000000
C	27.906209000000	50.447117000000	60.002688000000
C	26.718173000000	50.306492000000	59.086948000000
O	26.822562000000	50.308620000000	57.847150000000
H	28.589395000000	51.232505000000	59.637900000000
N	25.535643000000	50.085922000000	59.731702000000
C	24.423094000000	49.401851000000	59.075556000000
C	23.290712000000	50.340170000000	58.685934000000
C	24.026007000000	48.205442000000	59.953390000000
C	25.168651000000	47.185977000000	60.010036000000
N	25.312661000000	46.456305000000	61.124801000000
O	25.927127000000	47.044781000000	59.013253000000
H	19.505399000000	31.063580000000	60.034060000000
C	20.269342000000	30.781634000000	59.288822000000
C	21.368650000000	30.031277000000	60.036419000000
O	21.618786000000	30.270480000000	61.239343000000
C	20.804727000000	32.049581000000	58.562894000000
C	21.604658000000	32.978242000000	59.425668000000
C	22.920097000000	33.402418000000	59.337076000000
N	21.107838000000	33.601432000000	60.564065000000
C	22.083870000000	34.351257000000	61.126024000000
N	23.204329000000	34.248292000000	60.400117000000
N	22.079075000000	29.127903000000	59.313391000000
C	23.270480000000	28.443455000000	59.828627000000
H	23.031683000000	28.025476000000	60.823148000000
C	24.495500000000	29.369790000000	59.940420000000
C	24.916677000000	29.963466000000	58.594060000000
C	25.855795000000	31.152998000000	58.648938000000
O	26.012772000000	31.776189000000	59.757065000000
O	26.398429000000	31.489179000000	57.542220000000
H	25.342343000000	28.901557000000	65.956376000000
C	25.430583000000	29.593704000000	66.782123000000
H	25.235584000000	29.098263000000	67.755773000000
C	24.421510000000	30.757936000000	66.627331000000
C	24.388164000000	31.336776000000	65.246960000000
C	25.186823000000	32.266144000000	64.605428000000
N	23.483777000000	30.911236000000	64.279578000000
C	23.708249000000	31.541188000000	63.110943000000
N	24.745350000000	32.366895000000	63.296727000000
H	24.889820000000	37.055719000000	67.047362000000
C	25.452204000000	36.885366000000	66.121681000000
C	26.293394000000	38.157638000000	65.911573000000
O	25.820867000000	39.192165000000	65.401143000000
C	24.420851000000	36.607658000000	65.027445000000
C	24.958105000000	35.981022000000	63.751048000000
O	26.209652000000	35.665412000000	63.739972000000
O	24.148856000000	35.797594000000	62.792827000000
N	27.581711000000	38.125123000000	66.375820000000
C	28.490867000000	39.280094000000	66.368274000000
C	29.453417000000	39.381549000000	65.168195000000
O	30.168153000000	40.388168000000	65.026626000000
H	29.083429000000	39.257539000000	67.291201000000
N	29.441772000000	38.317835000000	64.334238000000
C	30.094837000000	38.263263000000	63.027507000000
C	29.278442000000	37.310481000000	62.129289000000
O	29.308215000000	37.416103000000	60.891992000000
C	31.567285000000	37.815043000000	63.110995000000
O	28.599659000000	36.417984000000	62.805419000000
O	24.281617000000	42.771560000000	62.767998000000
O	25.470773000000	39.062700000000	56.095248000000
O	24.822380000000	31.823702000000	54.801259000000
O	30.348157000000	30.678072000000	58.351380000000
O	26.298009000000	39.314742000000	61.779645000000
O	24.167346000000	40.057596000000	63.345712000000
O	25.581692000000	37.745645000000	53.705539000000
O	26.782416000000	43.929611000000	61.970590000000
O	28.073911000000	41.481025000000	61.660080000000
O	27.165249000000	35.558087000000	54.320739000000
H	30.699472000000	30.376337000000	66.032444000000
C	31.018906000000	30.890758000000	65.104316000000
H	31.997506000000	31.360958000000	65.227864000000
C	30.067346000000	31.992657000000	64.573214000000
C	28.921500000000	31.478823000000	63.674222000000
C	28.312533000000	32.534603000000	62.757180000000
O	28.101413000000	33.706470000000	63.219084000000
O	28.027100000000	32.165436000000	61.552482000000
H	36.469287000000	31.575154000000	63.532724000000
C	36.987893000000	32.139355000000	62.737355000000
H	37.705291000000	32.836251000000	63.183209000000
C	35.993754000000	32.856565000000	61.809202000000
C	35.173024000000	33.952869000000	62.510304000000
C	34.158955000000	34.674011000000	61.611836000000
N	33.050248000000	33.781261000000	61.242249000000
C	32.056867000000	34.080367000000	60.379650000000
N	32.021901000000	35.270952000000	59.756337000000
N	31.118021000000	33.161079000000	60.102701000000
O	23.991895000000	45.671523000000	63.479742000000
O	29.238869000000	29.787565000000	60.815742000000
O	25.986775000000	29.258848000000	55.023141000000
H	30.790691000000	28.048320000000	57.896952000000
H	28.296359000000	26.314142000000	58.505673000000
H	28.742925000000	27.802096000000	59.377827000000
H	32.830254000000	42.617840000000	54.064317000000
H	31.928741000000	40.512903000000	51.983610000000
H	31.491504000000	42.231138000000	51.903092000000
H	30.267207000000	43.284814000000	53.949358000000
H	30.640106000000	39.000294000000	53.402469000000
H	28.541069000000	42.913954000000	55.689426000000
H	28.894109000000	38.629207000000	55.135655000000
H	27.249758000000	41.470876000000	56.977600000000
H	32.925175000000	31.054143000000	53.642244000000
H	33.599339000000	30.053187000000	55.786386000000
H	31.822053000000	30.127245000000	55.613301000000
H	31.818977000000	31.190521000000	57.468834000000
H	32.858221000000	34.200090000000	54.955013000000
H	29.963404000000	34.402153000000	54.284504000000
H	30.211708000000	36.711128000000	55.521869000000
H	31.659939000000	36.015285000000	56.264988000000
H	32.487967000000	34.507724000000	52.521167000000
H	32.070559000000	37.027514000000	51.021789000000

H	33.018093000000	35.603622000000	50.516716000000
H	29.913452000000	35.728263000000	50.637956000000
H	30.879121000000	34.326398000000	50.066667000000
H	30.879067000000	35.854051000000	49.138911000000
H	22.268634000000	41.157120000000	60.629200000000
H	24.497110000000	40.429571000000	60.032432000000
H	24.218754000000	40.682316000000	58.296258000000
H	22.688678000000	38.812764000000	58.127437000000
H	22.415854000000	38.753303000000	59.878845000000
H	21.767515000000	43.025659000000	57.753636000000
H	23.249109000000	45.242063000000	59.095295000000
H	23.311271000000	44.612947000000	56.062227000000
H	24.040861000000	46.034402000000	56.836927000000
H	24.836839000000	42.198299000000	56.151017000000
H	26.163783000000	45.538750000000	58.462156000000
H	27.948369000000	43.665430000000	58.624585000000
H	27.638775000000	50.648837000000	61.053091000000
H	25.554068000000	50.079942000000	60.751387000000
H	24.852732000000	48.990953000000	58.143569000000
H	22.817604000000	50.808870000000	59.569694000000
H	22.506533000000	49.795346000000	58.129111000000
H	23.675614000000	51.144780000000	58.036195000000
H	23.148874000000	47.696502000000	59.508129000000
H	23.723465000000	48.524542000000	60.969354000000
H	25.990659000000	45.677508000000	61.162157000000
H	24.647507000000	46.499352000000	61.907162000000
H	19.781637000000	30.131664000000	58.539944000000
H	19.939345000000	32.587337000000	58.129099000000
H	21.445145000000	31.752083000000	57.713932000000
H	23.660766000000	33.168804000000	58.572467000000
H	21.964781000000	34.951820000000	62.028318000000
H	21.825353000000	28.982345000000	58.336953000000
H	23.475203000000	27.597496000000	59.149950000000
H	24.263806000000	30.179126000000	60.652311000000
H	25.331385000000	28.793919000000	60.379849000000
H	25.390752000000	29.203330000000	57.945700000000
H	24.030131000000	30.330242000000	58.035898000000
H	26.466936000000	29.974964000000	66.790959000000
H	23.403496000000	30.406017000000	66.878973000000
H	24.658702000000	31.562308000000	67.346557000000
H	26.024604000000	32.857580000000	64.974569000000
H	22.743332000000	30.222376000000	64.423224000000
H	23.120856000000	31.379427000000	62.201236000000
H	26.102755000000	36.011447000000	66.294437000000
H	23.637895000000	35.928165000000	65.413874000000
H	23.894659000000	37.536818000000	64.739547000000
H	27.904381000000	37.268969000000	66.826996000000
H	27.887705000000	40.204366000000	66.372397000000
H	28.775529000000	37.564104000000	64.519757000000
H	30.045767000000	39.266438000000	62.564494000000
H	31.645600000000	36.805343000000	63.550836000000
H	32.132657000000	38.522323000000	63.740798000000
H	32.021897000000	37.805272000000	62.105355000000
H	24.008419000000	42.856295000000	61.797646000000
H	23.853494000000	43.550580000000	63.187629000000
H	26.274512000000	39.653522000000	56.196184000000
H	25.282550000000	38.748426000000	57.020028000000
H	28.138394000000	39.536869000000	57.915625000000
H	25.254848000000	30.923219000000	54.861350000000
H	24.287488000000	31.797559000000	53.989852000000

H	29.700999000000	31.423429000000	58.472791000000
H	29.820670000000	30.014968000000	57.788905000000
H	29.272557000000	31.682106000000	55.168876000000
H	25.475596000000	39.516135000000	62.316062000000
H	26.289655000000	38.340440000000	61.618706000000
H	24.186180000000	41.051038000000	63.273305000000
H	24.640430000000	39.836588000000	64.186464000000
H	25.273199000000	38.380393000000	54.397359000000
H	25.176703000000	37.945529000000	52.836799000000
H	25.945979000000	43.529333000000	62.321157000000
H	27.326747000000	43.117599000000	61.771547000000
H	26.781730000000	37.665231000000	56.318775000000
H	27.478529000000	40.683135000000	61.664825000000
H	28.839039000000	41.219265000000	61.088144000000
H	26.745941000000	34.823130000000	54.867955000000
H	26.444445000000	36.179767000000	54.041072000000
H	31.155638000000	30.103665000000	64.337672000000
H	29.659862000000	32.595034000000	65.405821000000
H	30.675392000000	32.693561000000	63.970198000000
H	29.278406000000	30.655872000000	63.030668000000
H	28.090290000000	31.065711000000	64.281557000000
H	37.577246000000	31.399953000000	62.163503000000
H	36.541944000000	33.314509000000	60.961489000000
H	35.312850000000	32.107148000000	61.358447000000
H	34.640764000000	33.532164000000	63.388395000000
H	35.856661000000	34.723831000000	62.914446000000
H	33.759839000000	35.561532000000	62.142403000000
H	34.655400000000	35.028898000000	60.687083000000
H	31.239645000000	35.485198000000	59.113223000000
H	32.567493000000	36.051571000000	60.111169000000
H	30.250201000000	33.390609000000	59.597879000000
H	31.194905000000	32.216328000000	60.471136000000
H	24.808855000000	45.371600000000	63.953062000000
H	23.385375000000	45.958511000000	64.205676000000
H	29.732689000000	29.994481000000	59.981463000000
H	28.715388000000	30.606834000000	60.976974000000
H	26.393306000000	28.756539000000	55.809235000000
H	26.240778000000	28.714294000000	54.237006000000
H	28.975326000000	39.427786000000	59.272598000000
H	25.741611000000	33.052265000000	55.641060000000
H	28.494000000000	30.517326000000	56.010324000000
H	32.948833000000	32.918556000000	61.775150000000
H	27.437236000000	36.372971000000	55.739948000000
H	25.189908000000	32.984803000000	62.550545000000
H	20.165249000000	33.498200000000	60.940926000000
H	31.738140000000	38.519567000000	59.142521000000
H	30.537721000000	39.859967000000	57.347767000000
H	31.922496000000	40.961583000000	57.247799000000
H	31.451076000000	42.830059000000	58.271096000000
H	30.546194000000	42.978233000000	59.754082000000
H	33.169909000000	39.566185000000	58.953844000000
C	32.279198000000	39.175110000000	58.438897000000
C	31.376592000000	40.286166000000	57.929380000000
C	30.778675000000	41.068387000000	59.079418000000
N	30.928528000000	42.381353000000	59.019792000000
O	30.145390000000	40.510480000000	60.016069000000
H	32.630429000000	38.547651000000	57.600801000000
H	25.927145000000	34.248345000000	58.056783000000

S_1^E

Mn	24.815180524862	35.417874314678	60.829561851003
Mn	27.260157743733	35.135885464546	62.087809656309
Mn	27.243846252482	33.197499575868	60.064771147963
Mn	27.542511142928	33.095105975663	57.359687127742
Ca	27.751067638829	36.523813187975	59.000465292657
O	26.359115015952	36.411214014592	61.081827516116
O	28.280508017711	34.604033210596	60.695387872060
O	25.917057720216	33.956675457413	61.409972720164
O	28.472956434581	32.561518599237	58.869917670777
O	26.651219823860	34.158352861243	58.627503692027
O	26.358938413153	33.749447792597	55.795089896073
O	28.381625363001	31.706611752644	56.075864248434
O	27.294305447382	36.841294445886	56.600428917050
O	28.315786419693	38.856420419831	58.679862451513
H	30.386279030165	26.554955937319	57.103004959665
C	30.312281925810	27.064948105284	58.050497065358
H	30.925319004927	26.579769967580	58.850732973789
C	28.846218504196	27.298985554900	58.424648310271
C	28.135642118704	28.206688106379	57.411339262687
O	28.767522529729	29.257181858022	57.007477318456
O	26.970611596159	27.886671357742	57.024436370357
H	33.765459035897	42.205888982067	52.647135987357
C	33.048044850012	41.804450203878	53.345646070166
H	33.479306045702	40.971405958416	53.939089953862
C	31.762322155319	41.380888807820	52.605179790806
C	30.618042147472	41.122644314962	53.561683805925
C	30.025119757708	42.200237534966	54.255876304392
C	30.158725367381	39.823241853962	53.858564346826
C	29.046586997673	41.995388975587	55.237115231722
C	29.170411995940	39.599856275447	54.832192760439
C	28.619922475887	40.681467510891	55.556041036052
O	27.711679213896	40.445232596036	56.549089777656
H	33.875560957525	32.213250160731	54.592799962312
C	32.897071033905	31.686965782375	54.550602023566
C	31.697356994004	32.601135431110	54.504193252260
O	30.542983315651	32.148195818305	54.336856754609
C	32.783186143325	30.697312466429	55.731065466956
O	32.784709284415	31.384746481263	56.988579231662
N	31.948120524114	33.931509246895	54.646569932074
C	30.906064866082	34.937031734014	54.529380018810
C	30.959562465783	35.727570152545	53.207023544475
O	30.032892517821	36.530624932862	52.949111831575
C	30.802084021929	35.825582206409	55.778179467381
C	29.868325588552	35.258627645116	56.858012450019
O	29.035875049504	34.366845334440	56.493851084878
O	29.926370457950	35.763712808067	58.028739309031
N	31.987941636124	35.464485729741	52.369809872957
C	32.064647058917	35.924468018418	50.969467998593
C	30.860046906970	35.427153033636	50.158323013689
H	21.689023013936	40.969417012467	58.988050010459
C	22.547087984769	41.319580982541	59.569560993805
C	22.902398147900	42.778615683626	59.444581933089
O	23.703096048032	43.306274734862	60.256833743940
C	23.727119978169	40.368123085778	59.240681753423
C	23.170905727303	38.935319614380	59.104666301040
C	24.197064470528	37.819464994698	59.246104114242
O	25.262880806154	37.791779853410	58.581032173309
O	23.839266473696	36.900277380287	60.096675143620
N	22.355910079127	43.504153212818	58.440207360144
C	22.797167007942	44.863986011837	58.165327989058
H	21.965681996021	45.497421999082	57.900025002550
C	23.799379557869	44.946509427145	56.981195007852
C	25.035675245787	44.136465213266	57.184635384609
C	25.359536613567	42.857354633164	56.765904532615
N	26.091036339393	44.542149797120	57.986737697328
C	26.997859646914	43.546256832681	58.048696350694
N	26.575718829769	42.509575489434	57.318306186426
H	28.443634996014	49.488006998750	59.951731996405
C	27.906208997795	50.447117013977	60.002688001447
C	26.717473630130	50.305139017597	59.088577152575
O	26.820128115975	50.309414708070	57.848580853101
H	28.588585044600	51.232532303315	59.636553482911
N	25.536272954756	50.080711941634	59.734429850742
C	24.423094021695	49.401850985166	59.075556016005
C	23.290711980010	50.340170012477	58.685933997837
C	24.020880686078	48.201465886492	59.947274341152
C	25.151134193607	47.167453210115	59.992962554279
N	25.309080459553	46.450751735303	61.114479756702
O	25.887071525969	47.003046310382	58.982512711683
H	19.466062044022	31.135023826114	59.947581639543
C	20.259416081836	30.778629260406	59.267510388962
C	21.283885424811	30.027078941538	60.113323633976
O	21.492838117536	30.329722762937	61.309705607818
C	20.898042437920	31.975980240690	58.506142323185
C	21.644082596910	32.947060710327	59.370897165963
C	22.968990236430	33.352805908008	59.365690283956
N	21.062382447630	33.649727250523	60.419494038186
C	21.998313018942	34.427536910522	61.010737109058
N	23.173296769379	34.266959066287	60.390024424478
N	21.978489810960	29.049862655332	59.475532288393
C	23.160825790419	28.392365760540	60.045434746787
H	22.910184553866	28.037148820056	61.061112043655
C	24.390592292650	29.319288119303	60.104594896526
C	24.781012666659	29.878600001518	58.734587482381
C	25.706301115784	31.088886132235	58.713413934337
O	26.014366518389	31.644621928859	59.839780557193
O	26.092799654540	31.493269830711	57.580414700161
H	25.342342926025	28.901556975410	65.956376214179
C	25.430583169059	29.593704264511	66.782122492296
H	25.235583946491	29.098262816884	67.755773226508
C	24.423409381479	30.760622590819	66.624981449526
C	24.376565503800	31.340609691745	65.243621485394
C	25.172872963003	32.263689527438	64.589296017430
N	23.445161358497	30.933483818473	64.293490669836
C	23.651849758125	31.567240901953	63.123826319789
N	24.704271766841	32.377648015064	63.290441153770
H	24.889819994196	37.055719021315	67.047362053129
C	25.452203973247	36.885365939874	66.121680970011
C	26.290912803377	38.158652857224	65.910271215482
O	25.822871168007	39.188345176862	65.385295459400
C	24.414190760934	36.610779010992	65.033364998009
C	24.938726500263	35.980383431212	63.754225746699
O	26.177532812695	35.645712553959	63.722399187684
O	24.107306976818	35.810182226912	62.805146087356
N	27.572298180637	38.132518893556	66.391267241072
C	28.490867058020	39.280093994102	66.368273965833
C	29.449108561905	39.350355125489	65.161850373021
O	30.193522635959	40.335081753704	65.015910820407
H	29.083428967129	39.257539007890	67.291201018536

N	29.399839042239	38.289212576464	64.325499575086
C	30.066162507640	38.211210741941	63.026235625252
C	29.254845302383	37.253940727813	62.126137176823
O	29.288597028061	37.368793263215	60.888789591703
C	31.533322504949	37.751429237959	63.135743573869
O	28.582646567093	36.353796492100	62.794530956258
O	24.290091682025	42.754772402918	62.761870862170
O	25.497220895708	39.002800182512	56.098934626830
O	24.822381124689	31.823702135166	54.801258189673
O	30.493525487465	30.704590415317	58.392634852315
O	26.304198020804	39.281677920433	61.730413106630
O	24.183047127153	40.027009057160	63.311450886843
O	25.581691996310	37.745644929562	53.705538964567
O	26.780975998490	43.918501818302	61.952546434411
O	28.060069698716	41.465522899705	61.631885669212
O	27.413308612445	35.782305624319	54.075074518897
H	30.699472068594	30.376337072924	66.032444066037
C	31.018905840589	30.890757832143	65.104315794381
H	31.997506072516	31.360958068508	65.227864110767
C	30.070968240073	31.988710585857	64.572357345852
C	28.836171116725	31.464702020144	63.807496871999
C	28.229461759740	32.479278854168	62.842422522954
O	28.008887995877	33.669304761309	63.269235401664
O	27.958539298071	32.078898804477	61.655540537073
H	36.469287001266	31.575154069932	63.532724106665
C	36.987892992689	32.139354835498	62.737354724652
H	37.705291014320	32.836251079620	63.183209144520
C	35.989368365395	32.875466132653	61.830779463463
C	35.233837789725	34.003522066738	62.553416199720
C	34.195887991147	34.733778242715	61.692866707591
N	33.057970064104	33.856421097753	61.384938435945
C	32.070909441183	34.134993948883	60.508713004931
N	32.055797023656	35.298283784064	59.837573381748
N	31.123569842824	33.213417440717	60.264022637375
O	23.991894940771	45.671522930887	63.479742236362
O	29.238868976815	29.787565006446	60.815742065292
O	25.986774965057	29.258847853237	55.023141094902
H	30.803045455037	28.042976502728	57.894191363678
H	28.282543211843	26.352749221520	58.501773892358
H	28.777652044026	27.807296831760	59.407459936035
H	32.819214340895	42.617285957456	54.060601277362
H	31.955899953477	40.480168080902	51.992344396618
H	31.479783295642	42.186503902287	51.898313952666
H	30.346780156129	43.228068748094	54.033893149757
H	30.575872097721	38.959552405689	53.323324361861
H	28.621052073984	42.855112574790	55.765953598369
H	28.820878010538	38.583396906383	55.052107986007
H	27.204004871677	41.474694812211	56.980787234375
H	32.893024707648	31.099389549422	53.611432877279
H	33.643093121777	29.997471371231	55.684992439443
H	31.859461316296	30.098598914504	55.611992525621
H	31.949284210578	31.142234118945	57.481146054271
H	32.895258249514	34.219494873318	54.896702245892
H	29.959421347503	34.365720008614	54.463807699062
H	30.371545615287	36.806081242168	55.498795484986
H	31.794039677868	36.035540945872	56.218215005771
H	32.635036490571	34.732443675545	52.663110595629
H	32.104527894412	37.029821480935	50.963859941320
H	33.017902334995	35.552286235808	50.560691586226
H	29.912860200162	35.737968350478	50.625947534029
H	30.879121049262	34.326397957615	50.066666988646
H	30.894099489498	35.848593107355	49.137125731654
H	22.267573474725	41.158109536192	60.629152296983
H	24.490317975739	40.414708798509	60.037684641366
H	24.220534879068	40.675397962206	58.301087272244
H	22.684704111027	38.816511804404	58.115318422285
H	22.390128836940	38.760002668919	59.864627742782
H	21.753374541177	43.031051746002	57.766617948419
H	23.255455752277	45.244750639886	59.090980541625
H	23.296763565725	44.598302944014	56.060380684461
H	24.051584417485	46.012052817846	56.825005314515
H	24.813968834497	42.178508211097	56.108531178529
H	26.152159492756	45.484313663772	58.464001753913
H	27.932604917315	43.593971468476	58.608179936558
H	27.639804859644	50.649320908514	61.053260839910
H	25.554253069416	50.074167240435	60.754119550902
H	24.852833911121	48.993273228099	58.142368879213
H	22.813467262514	50.804526043463	59.569781660885
H	22.509825122458	49.795963763773	58.124053974937
H	23.676505690776	51.148114298732	58.040846966602
H	23.136628444758	47.704742856163	59.502358621418
H	23.725946710053	48.516691561258	60.966604128317
H	25.978902621294	45.664593661017	61.148200634190
H	24.654659386407	46.505576699947	61.905207337456
H	19.786539732692	30.108364455800	58.526925911441
H	20.093001439887	32.501730183466	57.956516699016
H	21.603024663648	31.600134869507	57.743253414656
H	23.774372620864	33.062636147215	58.690018470512
H	21.809818369455	35.086218110642	61.859109481373
H	21.765356024705	28.876713897065	58.493814194592
H	23.365195570588	27.504553034475	59.422035334554
H	24.176497407241	30.149902559576	60.797377662051
H	25.233306975944	28.754915512418	60.545806726358
H	25.261403720236	29.106499156428	58.103341363022
H	23.878577953069	30.204578007591	58.179064260958
H	26.467658687352	29.973119089096	66.790500594536
H	23.406284072832	30.411538795391	66.884169883007
H	24.665623751823	31.566548172602	67.340950881761
H	26.024555872378	32.844732239463	64.942701544618
H	22.697876162728	30.254923518208	64.449805590257
H	23.040018634242	31.421254578250	62.227643554571
H	26.103446189118	36.011277671168	66.290853729569
H	23.631897549170	35.933599812231	65.425385252628
H	23.888137748423	37.541313840433	64.750168329396
H	27.893003687788	37.277587561602	66.846151810811
H	27.897390963353	40.210529576276	66.362687572175
H	28.724049833492	37.545315650831	64.515906134659
H	30.031713793574	39.209572872048	62.552030046701
H	31.595672749531	36.747453991045	63.591065562347
H	32.097588860752	38.462234568280	63.762597542196
H	32.000154705019	37.722748013980	62.136155364476
H	24.015383182665	42.851006690601	61.793250604459
H	23.856693405647	43.522862174491	63.194945234520
H	26.308358297479	39.587387158927	56.215528323330
H	25.271154085846	38.700481979688	57.021490785552
H	28.133597755948	39.449751575348	57.897665796885
H	25.243125918512	30.916518147481	54.881062494638
H	24.287487738421	31.797558898736	53.989852071967
H	29.858035171692	31.466373361835	58.486617327168
H	29.935539798772	30.042815599417	57.863470816952

H	29.203198725160	31.917375921952	55.556831986261
H	25.483398958918	39.476744400288	62.270855997551
H	26.303884072292	38.307824569874	61.564973143357
H	24.200614214454	41.020455029026	63.246066213985
H	24.654696964423	39.802366343511	64.152597536226
H	25.300557555131	38.380656192486	54.411728250479
H	25.176702888082	37.945528980586	52.836799005317
H	25.947565998119	43.519870591604	62.312303135169
H	27.321968482014	43.104411962986	61.751961351710
H	26.667621760294	37.561236484405	56.333044463622
H	27.470650224365	40.663087341761	61.634269169656
H	28.833073110434	41.205568602399	61.068824815618
H	28.278476687845	36.040931504981	53.663927382562
H	26.762633430302	36.503579581442	53.824953260260
H	31.152748652572	30.103389532776	64.337224700944
H	29.754570455470	32.664205266828	65.388559719413
H	30.655946571849	32.619401920361	63.875141855505
H	29.103769352726	30.572878658387	63.215132861041
H	28.036210781336	31.156373405558	64.509799383473
H	37.569988430079	31.402107211376	62.153995091495
H	36.523237470220	33.309092615866	60.961410051001
H	35.268008132582	32.146744422597	61.410701343704
H	34.735393992656	33.610457687123	63.463682420934
H	35.956652925876	34.761719607503	62.910808178849
H	33.837591492829	35.637910576184	62.224862120460
H	34.657011782574	35.063083473207	60.741006144646
H	31.290069489370	35.503732189096	59.165595192613
H	32.609694245586	36.083367999176	60.169074184168
H	30.238264925218	33.456542377663	59.804907539073
H	31.180758239232	32.289012559440	60.684066390140
H	24.808855070974	45.371600039887	63.953061889411
H	23.385374979956	45.958511034407	64.205675895558
H	29.747072663456	30.052038925438	60.008906549563
H	28.710448402362	30.590780913051	61.039777351377
H	26.397551209529	28.775302957747	55.821035637345
H	26.240777992346	28.714294068898	54.237006045042
H	28.962322051231	39.341134645874	59.253829106741
H	25.816454891076	33.029939717557	55.326538737190
H	28.416925133119	30.731134457514	56.362224258005
H	32.931229110736	33.031645609311	61.970165797626
H	27.466156716428	36.355870428971	55.746754943830
H	25.139519934402	32.983356810591	62.531788702869
H	20.090586621369	33.578884563055	60.721894819989
H	31.737673401130	38.520712442430	59.143156604265
H	30.537160390398	39.861103782374	57.349979308003
H	31.924648488190	40.961537308516	57.246965179915
H	31.461923244482	42.828386047955	58.277929482718
H	30.547527723292	42.978622753815	59.754488824295
H	33.169909225954	39.566185213767	58.953844208198
C	32.279197665689	39.175109646021	58.438896711104
C	31.378227283467	40.286811756585	57.928972614852
C	30.778675109127	41.068386971475	59.079418042697
N	30.928527948451	42.381353002997	59.019791954538
O	30.136603945292	40.505474366411	60.005827484313
H	32.630040716246	38.545834760396	57.602052738935
H	26.809177676237	34.303753593314	55.104279562792

S_1^F

Mn	24.984966000000	35.330418000000	60.649998000000
Mn	27.308194000000	35.193732000000	62.139720000000
Mn	27.228537000000	33.339560000000	60.079430000000
Mn	27.540356000000	33.143261000000	57.205734000000
Ca	27.737937000000	36.595295000000	59.044456000000
O	26.405429000000	36.449363000000	61.134092000000
O	28.319308000000	34.686597000000	60.709036000000
O	25.999609000000	33.954835000000	61.524860000000
O	28.412471000000	32.770238000000	58.764756000000
O	26.324877000000	34.426731000000	58.982108000000
O	26.342759000000	33.777428000000	55.689674000000
O	28.376413000000	31.606457000000	55.823779000000
O	27.268660000000	36.826640000000	56.618525000000
O	28.396271000000	38.866528000000	58.634005000000
H	30.386279000000	26.554956000000	57.103005000000
C	30.312282000000	27.064948000000	58.050497000000
H	30.925319000000	26.579770000000	58.850733000000
C	28.838781000000	27.274929000000	58.417046000000
C	28.116383000000	28.155501000000	57.387152000000
O	28.734780000000	29.202670000000	56.961695000000
O	26.951905000000	27.819648000000	57.005838000000
H	33.765459000000	42.205889000000	52.647136000000
C	33.048045000000	41.804450000000	53.345646000000
H	33.479306000000	40.971406000000	53.939090000000
C	31.766558000000	41.379741000000	52.598574000000
C	30.619762000000	41.121953000000	53.550965000000
C	30.025628000000	42.200974000000	54.241987000000
C	30.164172000000	39.822634000000	53.853184000000
C	29.051367000000	41.997080000000	55.227001000000
C	29.179672000000	39.600890000000	54.830739000000
C	28.628506000000	40.683026000000	55.556711000000
O	27.730115000000	40.453411000000	56.553405000000
H	33.875561000000	32.213250000000	54.592800000000
C	32.897071000000	31.686966000000	54.550602000000
C	31.699743000000	32.591897000000	54.416442000000
O	30.571820000000	32.137358000000	54.124516000000
C	32.734615000000	30.748645000000	55.769489000000
O	32.649831000000	31.485728000000	56.994706000000
N	31.914447000000	33.918131000000	54.640367000000
C	30.856014000000	34.903514000000	54.507557000000
C	30.929035000000	35.716610000000	53.195860000000
O	29.997357000000	36.512116000000	52.933630000000
C	30.707338000000	35.776666000000	55.763155000000
C	29.779144000000	35.207959000000	56.841972000000
O	28.922595000000	34.329959000000	56.447202000000
O	29.840084000000	35.677557000000	58.013412000000
N	31.973376000000	35.470218000000	52.373797000000
C	32.064647000000	35.924468000000	50.969468000000
C	30.860047000000	35.427153000000	50.158323000000
H	21.689023000000	40.969417000000	58.988050000000
C	22.547088000000	41.319581000000	59.569561000000
C	22.913617000000	42.774565000000	59.433989000000
O	23.736295000000	43.294538000000	60.229002000000
C	23.726425000000	40.366770000000	59.246862000000
C	23.209414000000	38.918865000000	59.253009000000
C	24.275119000000	37.829448000000	59.265561000000
O	25.358187000000	37.931956000000	58.636992000000
O	23.930094000000	36.784858000000	59.959904000000
N	22.354451000000	43.505102000000	58.440309000000
C	22.797167000000	44.863986000000	58.165328000000

H	21.965682000000	45.497422000000	57.900025000000
C	23.799189000000	44.947485000000	56.981151000000
C	25.036257000000	44.139632000000	57.186518000000
C	25.359956000000	42.860853000000	56.769040000000
N	26.090788000000	44.548989000000	57.988598000000
C	27.000208000000	43.558839000000	58.055549000000
N	26.575913000000	42.521353000000	57.326064000000
H	28.443635000000	49.488007000000	59.951732000000
C	27.906209000000	50.447117000000	60.002688000000
C	26.717798000000	50.304299000000	59.088330000000
O	26.820963000000	50.306152000000	57.848365000000
H	28.588523000000	51.232523000000	59.636437000000
N	25.536430000000	50.080575000000	59.734073000000
C	24.423094000000	49.401851000000	59.075556000000
C	23.290712000000	50.340170000000	58.685934000000
C	24.020162000000	48.202222000000	59.948147000000
C	25.149472000000	47.167733000000	59.994065000000
N	25.310665000000	46.453718000000	61.116283000000
O	25.882502000000	47.000717000000	58.981292000000
H	19.548683000000	31.059471000000	60.002497000000
C	20.291340000000	30.762956000000	59.241515000000
C	21.388609000000	29.983422000000	59.963232000000
O	21.654268000000	30.189582000000	61.168480000000
C	20.842272000000	32.020527000000	58.508714000000
C	21.675148000000	32.928559000000	59.362948000000
C	22.988320000000	33.356400000000	59.244236000000
N	21.214242000000	33.522128000000	60.531650000000
C	22.209430000000	34.257111000000	61.081907000000
N	23.305318000000	34.172896000000	60.319555000000
N	22.080433000000	29.090093000000	59.209301000000
C	23.292288000000	28.408033000000	59.679095000000
H	23.075694000000	27.934494000000	60.653806000000
C	24.494430000000	29.359421000000	59.823662000000
C	24.867373000000	30.052498000000	58.510781000000
C	25.769141000000	31.266973000000	58.631708000000
O	26.054181000000	31.730142000000	59.783387000000
O	26.172268000000	31.773128000000	57.522253000000
H	25.342343000000	28.901557000000	65.956376000000
C	25.430583000000	29.593704000000	66.782123000000
H	25.235584000000	29.098263000000	67.755773000000
C	24.419579000000	30.756026000000	66.622968000000
C	24.389337000000	31.330329000000	65.240508000000
C	25.175158000000	32.276105000000	64.607065000000
N	23.511283000000	30.878308000000	64.260764000000
C	23.740816000000	31.508301000000	63.092532000000
N	24.753764000000	32.360245000000	63.291241000000
H	24.889820000000	37.055719000000	67.047362000000
C	25.452204000000	36.885366000000	66.121681000000
C	26.292748000000	38.158235000000	65.914131000000
O	25.817614000000	39.196940000000	65.413268000000
C	24.427844000000	36.605073000000	65.017144000000
C	24.972964000000	35.974321000000	63.738268000000
O	26.228971000000	35.666606000000	63.748673000000
O	24.175551000000	35.780189000000	62.777156000000
N	27.583721000000	38.124090000000	66.371607000000
C	28.490867000000	39.280094000000	66.368274000000
C	29.457172000000	39.388832000000	65.172652000000
O	30.165532000000	40.401339000000	65.035135000000
H	29.083429000000	39.257539000000	67.291201000000
N	29.459107000000	38.324296000000	64.341542000000
C	30.117227000000	38.270804000000	63.037432000000
C	29.306621000000	37.307569000000	62.142586000000
O	29.335648000000	37.417039000000	60.904142000000
C	31.592144000000	37.833825000000	63.129544000000
O	28.639146000000	36.413198000000	62.821923000000
O	24.295389000000	42.778425000000	62.752153000000
O	25.549935000000	39.018284000000	56.095116000000
O	24.822381000000	31.823702000000	54.801258000000
O	30.342317000000	30.754535000000	58.357229000000
O	26.333247000000	39.290571000000	61.767431000000
O	24.196948000000	40.049257000000	63.326296000000
O	25.581692000000	37.745645000000	53.705539000000
O	26.791549000000	43.932448000000	61.957694000000
O	28.067681000000	41.479853000000	61.635279000000
O	27.385951000000	35.788671000000	54.065345000000
H	30.699472000000	30.376337000000	66.032444000000
C	31.018906000000	30.890758000000	65.104316000000
H	31.997506000000	31.360958000000	65.227864000000
C	30.076467000000	31.993497000000	64.579421000000
C	28.872212000000	31.479723000000	63.764863000000
C	28.299474000000	32.519591000000	62.808259000000
O	28.105781000000	33.703811000000	63.262192000000
O	28.036204000000	32.144700000000	61.609718000000
H	36.469287000000	31.575154000000	63.532724000000
C	36.987893000000	32.139355000000	62.737355000000
H	37.705291000000	32.836251000000	63.183209000000
C	35.990043000000	32.864807000000	61.820195000000
C	35.181858000000	33.958327000000	62.539325000000
C	34.169954000000	34.699853000000	61.656294000000
N	33.051831000000	33.822830000000	61.277994000000
C	32.076623000000	34.138031000000	60.401567000000
N	32.071453000000	35.331789000000	59.781884000000
N	31.135866000000	33.229868000000	60.092802000000
O	23.991895000000	45.671523000000	63.479742000000
O	29.238869000000	29.787565000000	60.815742000000
O	25.986775000000	29.258848000000	55.023141000000
H	30.789078000000	28.050101000000	57.897316000000
H	28.294395000000	26.318176000000	58.504275000000
H	28.755020000000	27.793173000000	59.393519000000
H	32.815841000000	42.617008000000	54.059620000000
H	31.964221000000	40.478919000000	51.987081000000
H	31.487568000000	42.185029000000	51.889858000000
H	30.344752000000	43.228547000000	54.014698000000
H	30.583361000000	38.958800000000	53.319553000000
H	28.625927000000	42.858403000000	55.753769000000
H	28.832497000000	38.584428000000	55.055150000000
H	27.179658000000	41.542673000000	57.018065000000
H	32.924071000000	31.051534000000	53.644097000000
H	33.608428000000	30.065075000000	55.799057000000
H	31.830610000000	30.126014000000	55.623219000000
H	31.807940000000	31.221380000000	57.464943000000
H	32.832315000000	34.210315000000	54.978634000000
H	29.923509000000	34.316750000000	54.397384000000
H	30.257891000000	36.748258000000	55.480328000000
H	31.686013000000	36.011721000000	56.219251000000
H	32.628354000000	34.750562000000	52.680896000000
H	32.110994000000	37.029487000000	50.958140000000
H	33.018059000000	35.543099000000	50.570111000000
H	29.912536000000	35.739469000000	50.623890000000
H	30.879121000000	34.326398000000	50.066667000000

H	30.895592000000	35.847577000000	49.136705000000
H	22.268680000000	41.168103000000	60.631169000000
H	24.532069000000	40.488429000000	59.992625000000
H	24.157880000000	40.614652000000	58.259956000000
H	22.574439000000	38.739014000000	58.360933000000
H	22.559439000000	38.748785000000	60.129591000000
H	21.732457000000	43.039682000000	57.779224000000
H	23.255486000000	45.245585000000	59.090643000000
H	23.297938000000	44.597587000000	56.060388000000
H	24.051040000000	46.012950000000	56.824505000000
H	24.819187000000	42.177062000000	56.113483000000
H	26.148448000000	45.494027000000	58.465749000000
H	27.935482000000	43.606313000000	58.613631000000
H	27.639603000000	50.649410000000	61.053175000000
H	25.553662000000	50.076156000000	60.753786000000
H	24.852502000000	48.992767000000	58.142472000000
H	22.813817000000	50.804866000000	59.569778000000
H	22.509589000000	49.795888000000	58.124465000000
H	23.676435000000	51.147854000000	58.040481000000
H	23.135557000000	47.705828000000	59.503561000000
H	23.725683000000	48.518252000000	60.967317000000
H	25.982182000000	45.668759000000	61.151091000000
H	24.658206000000	46.510505000000	61.908528000000
H	19.773778000000	30.126292000000	58.501027000000
H	19.981633000000	32.577367000000	58.089270000000
H	21.463006000000	31.711632000000	57.649113000000
H	23.710478000000	33.153046000000	58.452933000000
H	22.121589000000	34.835044000000	62.002518000000
H	21.815354000000	28.976341000000	58.231760000000
H	23.508088000000	27.602134000000	58.956630000000
H	24.255191000000	30.113959000000	60.590626000000
H	25.355950000000	28.783029000000	60.208659000000
H	25.363975000000	29.349519000000	57.815652000000
H	23.960488000000	30.411101000000	57.983001000000
H	26.466504000000	29.976020000000	66.790121000000
H	23.401766000000	30.402734000000	66.873888000000
H	24.653736000000	31.563656000000	67.339442000000
H	25.993181000000	32.889644000000	64.984399000000
H	22.785324000000	30.172583000000	64.395397000000
H	23.173429000000	31.330603000000	62.173620000000
H	26.104448000000	36.012736000000	66.294520000000
H	23.643886000000	35.925425000000	65.401228000000
H	23.902329000000	37.534162000000	64.728144000000
H	27.909622000000	37.264269000000	66.813300000000
H	27.885294000000	40.202754000000	66.374229000000
H	28.793904000000	37.566995000000	64.518804000000
H	30.062561000000	39.272283000000	62.571702000000
H	31.675349000000	36.825741000000	63.572342000000
H	32.150872000000	38.546114000000	63.759836000000
H	32.051560000000	37.823989000000	62.126003000000
H	24.032623000000	42.855164000000	61.779307000000
H	23.857624000000	43.556841000000	63.163147000000
H	26.361179000000	39.613105000000	56.204773000000
H	25.305786000000	38.778863000000	57.029607000000
H	28.181860000000	39.473623000000	57.866753000000
H	25.255116000000	30.921154000000	54.870610000000
H	24.287488000000	31.797559000000	53.989852000000
H	29.695122000000	31.506644000000	58.454855000000
H	29.806441000000	30.074056000000	57.825360000000
H	29.206047000000	31.777745000000	55.311214000000
H	25.507497000000	39.488227000000	62.298284000000
H	26.337455000000	38.313730000000	61.611081000000
H	24.211637000000	41.042105000000	63.260389000000
H	24.658839000000	39.824282000000	64.172719000000
H	25.313038000000	38.388411000000	54.409538000000
H	25.176703000000	37.945529000000	52.836799000000
H	25.956018000000	43.533890000000	62.313233000000
H	27.331132000000	43.117720000000	61.755389000000
H	26.663211000000	37.570752000000	56.359453000000
H	27.484124000000	40.672490000000	61.654118000000
H	28.838565000000	41.216753000000	61.071248000000
H	28.251968000000	36.032341000000	53.645869000000
H	26.739420000000	36.517933000000	53.820571000000
H	31.151691000000	30.105123000000	64.335406000000
H	29.731494000000	32.643331000000	65.404636000000
H	30.673366000000	32.649098000000	63.916364000000
H	29.164092000000	30.601465000000	63.163676000000
H	28.048526000000	31.153673000000	64.431174000000
H	37.573956000000	31.401480000000	62.158475000000
H	36.533284000000	33.327256000000	60.971683000000
H	35.301521000000	32.122179000000	61.369968000000
H	34.650971000000	33.530357000000	63.414720000000
H	35.874026000000	34.718090000000	62.950311000000
H	33.781345000000	35.583997000000	62.200275000000
H	34.664644000000	35.062312000000	60.733726000000
H	31.302696000000	35.557072000000	59.126520000000
H	32.596707000000	36.109475000000	60.172491000000
H	30.253253000000	33.500340000000	59.640365000000
H	31.176711000000	32.290848000000	60.482009000000
H	24.808855000000	45.371600000000	63.953062000000
H	23.385375000000	45.958511000000	64.205676000000
H	29.720535000000	30.041984000000	59.988182000000
H	28.731943000000	30.605784000000	61.041977000000
H	26.389971000000	28.753699000000	55.812034000000
H	26.240778000000	28.714294000000	54.237006000000
H	29.017024000000	39.361667000000	59.228279000000
H	25.797295000000	33.039156000000	55.244408000000
H	28.407310000000	30.652966000000	56.146094000000
H	32.922885000000	32.969616000000	61.820275000000
H	27.432687000000	36.356305000000	55.756749000000
H	25.207027000000	32.982126000000	62.549251000000
H	20.284496000000	33.407343000000	60.935230000000
H	31.736298000000	38.521476000000	59.142719000000
H	30.541042000000	39.864847000000	57.345626000000
H	31.929351000000	40.964869000000	57.250583000000
H	31.460247000000	42.828893000000	58.277021000000
H	30.549014000000	42.978216000000	59.755609000000
H	33.169909000000	39.566185000000	58.953844000000
C	32.279198000000	39.175110000000	58.438897000000

C	31.380148000000	40.288489000000	57.928612000000
C	30.778675000000	41.068387000000	59.079418000000
N	30.928528000000	42.381353000000	59.019792000000
O	30.140886000000	40.507073000000	60.009357000000
H	32.630480000000	38.546144000000	57.601922000000
H	26.764557000000	34.353391000000	54.991964000000
